# Boosting Ligand-Mediated Hepatic mRNA Delivery and Gene Editing Through Coordinated Pairing of Polyethylene Glycol-Modified Lipids

**DOI:** 10.1021/acsnano.6c10855

**Published:** 2026-09-08

**Authors:** Saed Abbasi, Kimberly M. Bockley, Johannes Dohr, Emily Chen, Davell M. Carter, Cynthia A. Berlinicke, Xizhen Lian, Maria Viskadourou, Emma Burke, Laura M. Ensign, Gregory A. Newby, Justin Hanes

**Affiliations:** † Center for Nanomedicine at the Wilmer Eye Institute, Johns Hopkins University School of Medicine, Baltimore, Maryland 21231, United States; ‡ Department of Ophthalmology, Johns Hopkins University School of Medicine, Baltimore, Maryland 21231, United States; § Department of Physiology, Pharmacology & Therapeutics, Johns Hopkins University School of Medicine, Baltimore, Maryland 21231, United States; ∥ Department of Genetic Medicine, Johns Hopkins University, Baltimore, Maryland 21205, United States; ⊥ Department of Chemical and Biomolecular Engineering, Johns Hopkins University, Baltimore, Maryland 21231, United States; # Department of Materials Science and Engineering, Johns Hopkins University, Baltimore, Maryland 21231, United States; ¶ Institute for NanoBioTechnology, Johns Hopkins University, Baltimore, Maryland 21231, United States; ∇ Department of Pediatrics, Johns Hopkins University School of Medicine, Baltimore, Maryland 21231, United States; ○ Department of Biomedical Engineering, 1466 Johns Hopkins University, Baltimore, Maryland 21231, United States

**Keywords:** lipid nanoparticle, nanomedicine, base editing, active targeting, nucleic acid therapeutics, cardiovascular disease, liver disease

## Abstract

Active targeting can improve the efficacy of mRNA therapeutics, but ligand-mediated targeting with lipid nanoparticles (LNPs) remains challenging. Here, we studied the impact of polyethylene glycol (PEG)-lipid composition and triantennary *N*-acetylgalactosamine (TriGalNAc)-modification on the hepatic delivery efficiency of mRNA-LNPs. Incorporating TriGalNAc-lipid into LNPs containing dimyristoyl glycerol (DMG)-PEG2000 or distearoylphosphatidylethanolamine (DSPE)-PEG2000, excipients used in FDA-approved RNA-LNPs or liposomes, did not further improve hepatic mRNA delivery. We discovered that incorporating TriGalNAc-lipid into LNPs containing DSPE-PEG350 increased hepatic luciferase expression 4–6-fold over TriGalNAc-conjugated or unconjugated DMG-PEG2000 LNPs, and 60-fold over TriGalNAc-conjugated or unconjugated DSPE-PEG2000 LNPs in vivo. Despite its relatively low molecular weight PEG, unconjugated DSPE-PEG350 LNP showed moderately extended blood circulation but only moderately reduced cellular uptake in vitro, potentially providing a balanced platform for adding TriGalNAc ligands and improving the overall hepatic delivery efficiency in vivo. The pairing of TriGalNAc-lipid and DSPE-PEG350 in LNPs containing adenine base editor RNAs against PCSK9 lowered plasma cholesterol in wild-type mice, while conventional DMG-PEG2000 LNP was ineffective at the same dose. This study highlights the importance of redesigning the LNP surface to potentiate ligand-mediated active targeting to the liver.

## Graphical abstract

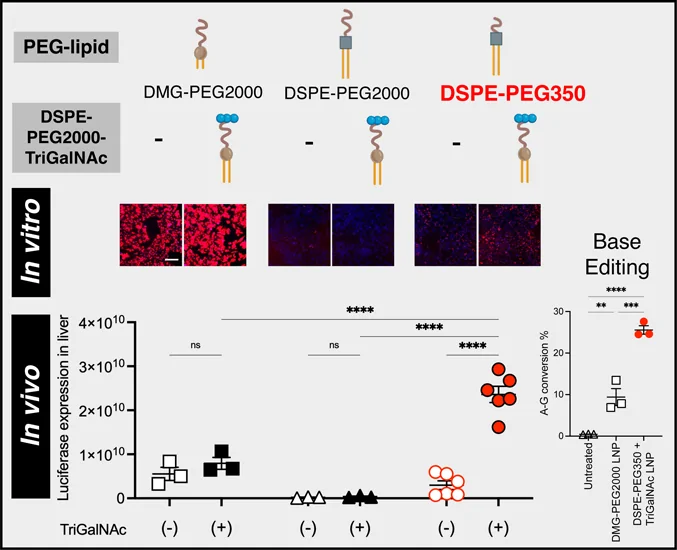

## 1 Introduction

Genetic defects in the liver can lead to a range of diseases that affect metabolism, blood function, or the cardiovascular system.^1,2^ Hepatocytes comprise 70–80% of the liver’s mass and control many metabolic and synthetic functions.^3^ Recent advancements in gene therapies, particularly the use of lipid nanoparticles (LNPs) and mRNA encoding gene editors, have facilitated hepatic gene editing through a unique strategy that targets hepatocytes more specifically.^4,5^ mRNA-LNPs typically comprise four primary lipid components: an ionizable cationic lipid, cholesterol, a helper phospholipid, and a polyethylene glycol (PEG) lipid stabilizer.^6,7^ After intravenous administration, the LNP surface adsorbs blood proteins, including apolipoprotein E (ApoE), leading to spontaneous PEG-lipid desorption.^8^ Consequently, the protein corona matures and drives LNP uptake by the low-density lipoprotein receptors (LDLRs) expressed on the surface of hepatocytes.^9^ Although mRNA-LNP delivery of gene editors has demonstrated significant promise in clinical trials,^10,11^ LNP-related adverse events may limit the maximum tolerated dose.^12,13^ Recently, LNP-associated adverse events led to the discontinuation of a Phase 1b clinical trial of an adenine base editor (ABE) mRNA-LNP developed for the treatment of hypercholesterolemia.^14^ Therefore, a more potent liver-targeting strategy is needed to enhance hepatic gene editing efficiency and reduce dose-limiting toxicities.

Active targeting using the triantennary *N*-acetylgalactosamine (TriGalNAc) ligand has long been investigated for the delivery of nanoparticles into hepatocytes via the asialoglycoprotein receptor (ASGPR) pathway.^15^ TriGalNAc has been functionalized on the surface of nucleic acid LNPs,^16^ liposomes,^17^ polymeric nanoparticles,^18^ or ribonucleoproteins.^19^ Targeting hepatocytes via the ASGPR is promising for treating conditions characterized by LDLR deficiency, such as homozygous familial hypercholesterolemia, where LNP uptake by the LDLR is reduced.^20^ A recent study optimized the TriGalNAc conjugated-lipid structure and found that the incorporation of TriGalNAc lipid bearing a 36-unit PEG spacer and two saturated C18 lipid chains into the mRNA-LNP as a fifth component induced strong hepatic base editing in LDLR knockout mice.^21^ However, in wild-type mice, both the TriGalNAc-conjugated and unconjugated LNPs induced indistinguishable levels of base editing efficiency, which were as efficient as those induced by the TriGalNAc modification in LDLR knockout mice.^21^ While TriGalNAc conjugation offered an alternative strategy for potentiating mRNA uptake by the ASGPR pathway in the absence of LDLR expression in that study,^21^ the current LNP formulation design strategies have yet to realize the full potential of ligand-based active targeting for enhancing the overall delivery efficiency, including delivery to wild-type animals.

Extensive efforts have been dedicated to understanding the influence of lipid composition and structure on mRNA delivery efficiency.^22,23^ PEG-lipid plays a significant role in controlling the colloidal stability, blood circulation, and postdelivery events, including cellular uptake and endosomal escape.^24,25^ LNPs containing dimyristoyl glycerol (DMG)-PEG2000, an excipient used in FDA-approved LNPs such as Onpattro and Spikevax, can undergo rapid elimination from circulation.^8^ Approaches that produce stable LNPs through the incorporation of distearoylphosphatidylethanolamine (DSPE)-PEG2000, an excipient used in FDA-approved stealth liposomes such as Doxil, can reduce cellular uptake and compromise intracellular trafficking.^26,27^ Here, we studied the effects of stabilizer PEG-lipid structure, including PEG molecular weight, lipid chain structure, and concentrations on the synergistic enhancement in hepatic mRNA delivery imparted by the addition of DSPE-PEG2000-TriGalNAc, with the aim of improving the efficiency of in vivo hepatic base editing in wild-type mice.

In this study, while the incorporation of DSPE-PEG2000-TriGalNAc into DMG-PEG2000 or DSPE-PEG2000 LNPs did not further improve the hepatic mRNA delivery compared to unconjugated counterparts following intravenous injections, we found that incorporating DSPE-PEG2000-TriGalNAc into LNPs containing a PEG-lipid comprising a low Mw PEG (350–500 g/mol) induced up to 9-fold increase in hepatic mRNA delivery compared to unconjugated counterparts. Particularly, we discovered that pairing of DSPE-PEG2000-TriGalNAc and DSPE-PEG350 achieved a 4–6-fold increase in liver protein expression compared to TriGalNAc-conjugated or unconjugated DMG-PEG2000 LNPs, and a 60-fold increase compared to TriGalNAc-conjugated or unconjugated DSPE-PEG2000 LNPs in wild-type mice. We unexpectedly found that unconjugated DSPE-PEG350 LNP induced a moderate increase in circulation time while only moderately reducing LNP uptake compared to the Onpattro-like, DMG-PEG2000 LNP, thereby potentially serving as a platform to enable active targeting after the addition of targeting ligands. The LNP containing DSPE-PEG350 paired with DSPE-PEG2000-TriGalNAc led to substantial base editing in the mouse liver at relatively low mRNA doses, surpassing the editing efficiency of the DMG-PEG2000 LNP. This approach could provide more potent gene editing in the liver with improved safety, broadening the therapeutic options available for a range of diseases affecting the liver.

## 2 Results

### 2.1 Incorporation of DSPE-PEG2000-TriGalNAc in LNPs Based on DMG-PEG2000 Fails to Improve Hepatic mRNA Delivery Efficiency

LNPs with composition similar to the FDA-approved Onpattro formulation were prepared by mixing an ionizable cationic lipid (DLin-MC3-DMA; abbreviated as MC3), cholesterol, a phospholipid (DSPC), and a PEG-lipid (1.5 mol % DMG-PEG2000), according to previous reports.^28,29^ A table summarizing the lipid compositions and molar ratios used in this study listed in Table S1. TriGalNAc-conjugated LNP (LNP 2) was prepared by adding DSPE-PEG2000-TriGalNAc to LNP 1 as a fifth component (Figure 1a). The unconjugated (LNP 1) and conjugated (LNP 2) LNPs based on DMG-PEG2000 showed comparable particle size and polydispersity index (PDI) characteristics (Table S1). Both LNP 1 and LNP 2 showed high luciferase expression in the liver 4 and 24 h postinjection by in vivo luciferase imaging (Figure 1b) and in the extracted livers after sacrifice at 24 h (Figure 1c). However, the addition of DSPE-PEG2000-TriGalNAc in LNP 2 did not further enhance luciferase expression in the liver compared to unconjugated LNP (LNP 1) (Figure 1b,c). A representative IVIS image from one mouse per treatment group is provided in Figure 1 and all IVIS images from all replicates are provided in Figure S1.

**Figure 1:**
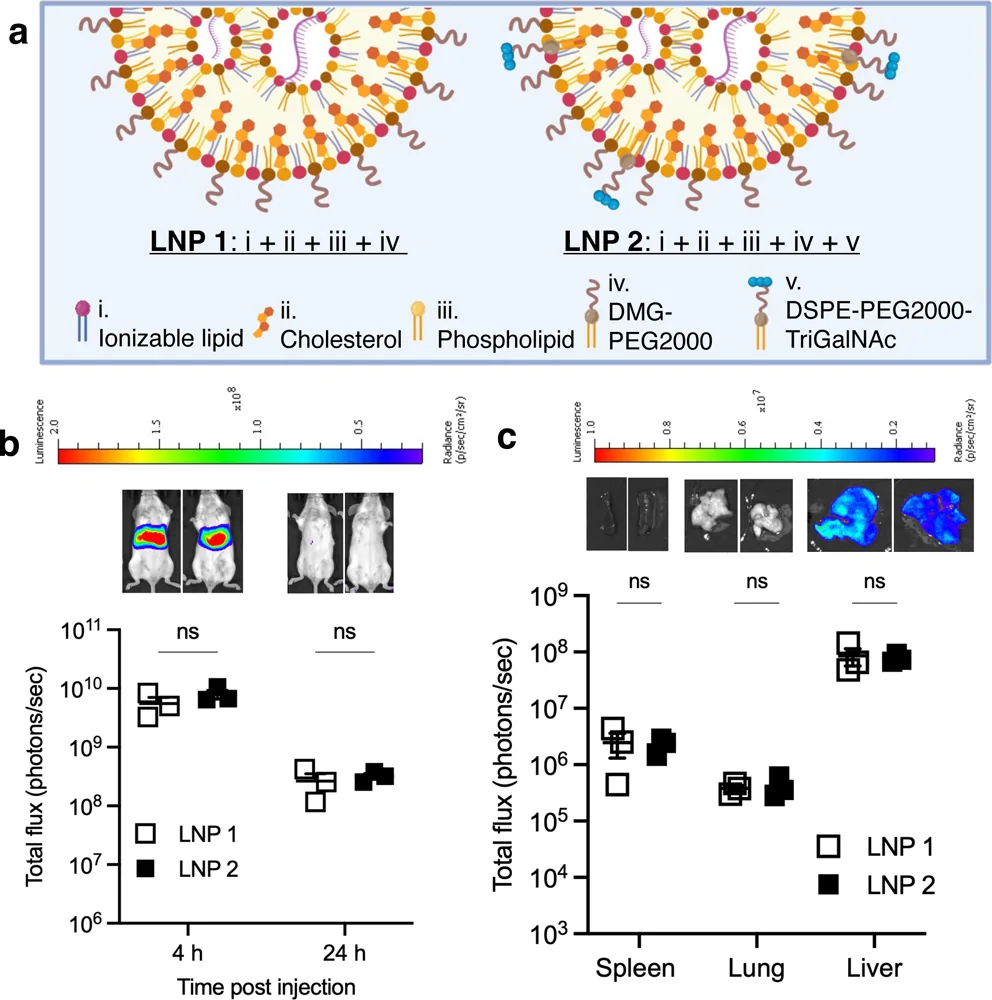
Incorporation of DSPE-PEG2000-TriGalNAc in LNPs based on DMG-PEG2000 fails to improve hepatic mRNA delivery efficiency. (a) Compositions of unconjugated LNP containing 0 mol % DSPE-PEG2000-TriGalNAc (LNP 1) and TriGalNAc-conjugated LNP containing 0.1 mol % DSPE-PEG2000-TriGalNAc (LNP 2). Both LNPs 1 and 2 contain 1.5 mol % DMG-PEG2000 (b) Liver luciferase expression 4 and 24 h post intravenous injection of 7.5 μg fLuc mRNA in BALB/c mice, as quantified by whole-body imaging using IVIS. (c) Luciferase expression in extracted organs 24 h postinjection. The data represent the mean ± SEM (*n* = 3 mice). Statistical significance was calculated using two-tailed, unpaired *t*-test. ns: nonsignificant. *P < 0.05. **P < 0.01. ***P < 0.001. ****P < 0.0001. Representative IVIS images are shown. Images from all replicates are available in Figure S1.

### 2.2 Studying the PEG-Lipid Structure–Activity Relationship Identifies Conditions for Improving Ligand-Mediated Hepatic mRNA Delivery In Vivo

Here, we studied the impact of PEG-lipid variants on the liver-targeting effect of TriGalNAc-lipid-modified mRNA-LNPs (Figure 2a). Since the incorporation of PEG-lipids can improve particle stability and provide a stealth effect after intravenous administration, we increased the PEG-lipid concentration to 3 mol % in all formulations in Figure 2. We evaluated in vivo luciferase expression in the liver 4 h (Figure 2b,e,h,k) and 24 h (Figure 2c,f,i,l) post intravenous injection, and then ex vivo luciferase biodistribution in the extracted organs at 24 h after sacrifice (Figure 2d,g,j,m). All tested LNPs exhibited the highest luciferase expression 4 h post injection, which was then reduced at 24 h (Figure 2). The luciferase expression in the extracted livers at 24 h post injection was used to quantitatively compare the delivery efficiency of various formulations.

**Figure 2:**
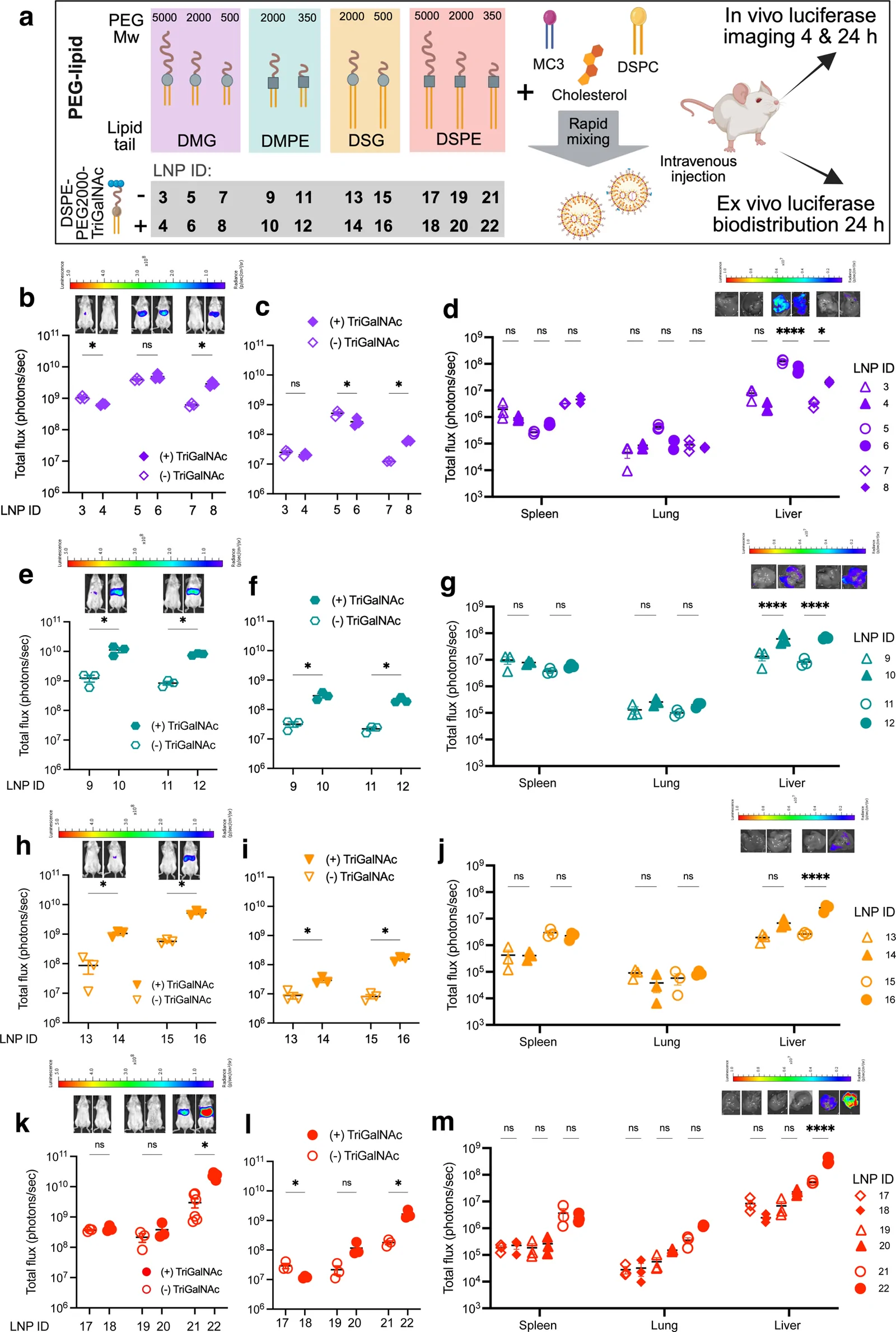
The impact of PEG-lipid choice on hepatic luciferase expression induced by incorporation of DSPE-PEG2000-TriGalNAc in mRNA-LNPs. (a) Illustration showing the different types of PEG-lipids used in formulating TriGalNAc-conjugated and unconjugated LNPs for intravenous administration. (b,e,h,k) Liver luciferase expression 4 h post intravenous injection of 7.5 μg fLuc mRNA in BALB/c mice, as quantified by whole-body imaging using IVIS. The data represent the mean ± SEM (*n* = 3 mice, except for LNPs 21 and 22, *n* = 6). (c,f,i,l) Liver luciferase expression 24 h post intravenous injection, as quantified by whole-body imaging. The data represent the mean ± SEM (*n* = 3 mice). Statistical significance was calculated using two-tailed, unpaired *t* tests between the unconjugated and conjugated LNPs. ns: nonsignificant. *P < 0.05. (d,g,j,m) Ex vivo luciferase expression in the extracted organs 24 h postinjection. The data represent the mean ± SEM (*n* = 3 mice). Statistical significance was calculated using two-way ANOVA followed by Tukey’s posthoc analysis. ns: nonsignificant. *P < 0.05. ****P < 0.0001. (b–d) Effect of modifying LNPs with 3 mol % DMG-PEG variants with different PEG chain lengths, with or without 0.1 mol % DSPE-PEG2000-TriGalNAc (LNPs 3–8). (e–g) Effect of modifying LNPs with 3 mol % DMPE-PEG variants with different PEG chain lengths, with or without 0.1 mol % DSPE-PEG2000-TriGalNAc (LNPs 9–12) (h–j) Effect of modifying LNPs with 3 mol % DSG-PEG variants with different PEG chain lengths, with or without 0.1 mol % DSPE-PEG2000-TriGalNAc (LNPs 13–16). (k–m) Effect of modifying LNPs with 3 mol % DSPE-PEG variants with different PEG chain lengths, with or without 0.1 mol % DSPE-PEG2000-TriGalNAc (LNPs 17–22). Representative IVIS images are shown. Images from all replicates are available in Figure S1.

First, we tested the impact of the PEG length (500, 2000, and 5000 g/mol) of C14, glyceride-based PEG-lipids (DMG-PEG). LNP 5, containing DMG-PEG2000, an excipient used in FDA-approved RNA-LNP products, was used as a benchmark for comparisons. Among the unconjugated LNPs (LNPs 3, 5, and 7), LNP 5 showed the highest luciferase expression in the extracted livers, which was 16- and 40-fold higher than that of LNPs 3 and 7, respectively (Figure 2d). While the incorporation of DSPE-PEG2000-TriGalNAc into LNPs based on DMG-PEG2000 or DMG-PEG5000 did not increase luciferase expression in the liver compared to unconjugated LNPs (LNP 4 vs LNP 3; LNP 6 vs LNP 5, Figure 2b–d), the addition of DSPE-PEG2000-TriGalNAc into DMG-PEG500 LNP induced 6.5-fold enhancement in luciferase expression in the extracted livers compared to the unconjugated LNP (LNP 8 vs LNP 7, Figure 2d). Nonetheless, the liver expression induced by LNP 8 remained 6-fold lower than LNP 5 (Figure 2d). Notably, similar to what was observed at 1.5 mol % DMG-PEG2000 (LNPs 1 and 2; Figure 1), LNPs containing 3 mol % DMG-PEG2000 provided similar liver expression with and without the addition of DSPE-PEG2000-TriGalNAc (LNP 6 vs LNP 5; Figure 2b–d). This suggests that tuning the PEG-lipid concentration alone may not be sufficient to boost the TriGalNAc-mediated hepatic delivery efficiency.

We next evaluated the effect of C14, phospholipid-based PEG-lipid (DMPE-PEG) at 2 PEG lengths (350 and 2000 g/mol). Unconjugated LNPs containing DMPE-PEG2000 (LNP 9) and DMPE-PEG350 (LNP 11) showed a 9- and 15-fold lower luciferase expression in the extracted livers compared to LNP 5, respectively (Figure 2g). TriGalNAc-conjugated DMPE-PEG2000 LNP showed a 4.6-fold enhancement in the luciferase expression in the extracted livers compared to the unconjugated counterpart, while conjugated DMPE-PEG350 LNP showed an 8-fold enhancement compared to the unconjugated counterpart (LNP 10 vs LNP 9; LNP 12 vs LNP 11, Figure 2g). However, the highest luciferase expression in extracted livers among DMPE-PEG LNPs was observed with LNP 12, though it remained 2-fold lower than LNP 5 (Figure 2d,g).

We also studied the effect of C18, glyceride-based PEG lipid (DSG-PEG) at 2 PEG lengths (500 and 2000 g/mol). Unconjugated LNPs containing DSG-PEG2000 and DSG-PEG500 (LNPs 13 and 15, respectively) showed around 50–60-fold lower luciferase expression in the extracted livers compared to LNP 5 (Figure 2j). TriGalNAc-conjugated DSG-PEG2000 LNP showed a 3.5-fold enhancement in the luciferase expression in the livers compared to the unconjugated counterpart, while conjugated DSG-PEG500 LNP showed a 9.6-fold enhancement compared to the unconjugated counterpart (LNP 14 vs LNP 13; LNP 16 vs LNP 15, Figure 2j). However, the highest liver expression in the extracted livers among DSG-PEG LNPs was observed with LNP 16, though it remained 5-fold lower than LNP5 (Figure 2d,j).

Lastly, we evaluated the effect of C18, phospholipid-based PEG lipid (DSPE-PEG) at 3 PEG lengths (350, 2000, and 5000 g/mol) (Figure 2k–m). While unconjugated LNPs containing DSPE-PEG2000 and DSPE-PEG5000 (LNPs 17 and 19, respectively) showed 16–18-fold lower luciferase expression in the extracted livers compared to LNP 5, the luciferase expression induced by unconjugated DSPE-PEG350 LNP (LNP 21) was only 2-fold lower than that of LNP 5 (Figure 2m). Incorporating a DSPE-PEG2000-TriGalNAc into DSPE-PEG5000 or DSPE-PEG2000 LNPs did not enhance luciferase expression in the liver (LNP 18 vs LNP 17; LNP 20 vs LNP 19, respectively) (Figure 2j–m). Interestingly, the incorporation of a DSPE-PEG2000-TriGalNAc into DSPE-PEG350 LNP showed a 6.3-fold enhancement in liver expression compared to unconjugated LNP (LNP 22 vs LNP 21, Figure 2m). LNP 22 showed a high luciferase expression in extracted livers in Figure 2, which was 3–6-fold higher than TriGalNAc-conjugated or unconjugated DMG-PEG2000 LNPs (LNPs 6 and 5, respectively; Figure 2d,m). In addition, compared to the Onpattro-like LNP 1 (1.5 mol % DMG-PEG2000), LNP 22 induced a 4-fold higher luciferase expression in the extracted livers (Figures 1 and 2m).

We then plotted the relationship between either the PEG chain or lipid tail molecular weight and the synergistic enhancement of hepatic mRNA delivery conferred by incorporating DSPE-PEG2000-TriGalNAc (Figure S2). There was a statistically significant negative correlation between the PEG Mw of the PEG-lipid and the enhancement of hepatic luciferase expression conferred by DSPE-PEG2000-TriGalNAc (Figure S2a). In addition, no significant Spearman’s correlation was seen in the plot between the lipid tail Mw and fold-change in luciferase expression in the liver (Figure S2b). This suggests a nonmonotonic relationship, where pairing DSPE-PEG2000-TriGalNAc with an intermediate-Mw lipid tail (such as DMPE or DSG) of the PEG-lipid seemed to produce higher luciferase expression in the liver relative to the unconjugated counterparts, regardless of the PEG chain length (Figure S2b). Although LNP 22 showed the lowest fold enhancement in hepatic delivery efficiency compared to its unconjugated counterpart in the low Mw PEG group (Figure S2), it achieved the highest luciferase expression in the liver among LNPs 1–22 (Figures 1 and 2).

We further confirmed that LNP 22 exhibited greater liver accumulation 1 h postinjection compared to LNP 1 or LNP 21, as demonstrated by tracking Cy5-labeled mRNA biodistribution (Figure S3). Interestingly, LNP 21 showed lower liver accumulation than LNP 22, but higher accumulation in other organs including the lung and spleen. Furthermore, we confirmed that the enhancement in hepatic mRNA delivery conferred by LNP 22 occurs on the level hepatocytes, because hepatocytes of livers treated with LNP 22 provided higher mCherry expression relative to the untreated or LNPs 1 and 21- treated groups after intravenous injections of mCherry mRNA-LNPs (Figure S4). Please note that the low detection sensitivity of mCherry fluorescence intensity by flow cytometry resulted in the inability to distinguish the mCherry expression levels induced by LNPs 1 and 21 relative to that of the untreated group due to the high background level (Figure S4). Collectively, these data support that substituting the conventional DMG-PEG2000 with DSPE-PEG350 enhances the hepatic mRNA delivery indued by DSPE-PEG2000-TriGalNAc.

To further clarify the role of PEG ligand spacer length on the hepatic targeting efficiency, we formulated LNPs containing 0.1 mol % DSPE-PEG350-TriGalNAc along with either DSPE-PEG350 or DMG-PEG2000 as the stabilizer PEG-lipids (Figure S5). The hepatic luciferase expression was indisngushable when DSPE-PEG350-TriGalNAc was paired with either DMG-PEG2000 or DSPE-PEG350 (Figure S5). In contrast, the pairing of DSPE-PEG2000-TriGalNAc with DSPE-PEG350 induced a 3-fold increase in hepatic luciferase expression compared to its pairing with DMG-PEG2000 (Figure S5). This suggests that to enhance the TriGalNAc-mediated hepatic delivery in vivo, the short stabilizer PEG (DSPE-PEG350) should be paired with a longer ligand spacer PEG (DSPE-PEG2000-TriGalNAc).

### 2.3 Studying Cellular Uptake and Luciferase Expression In Vitro Reveals Critical Deviations from the In Vivo Condition

We next evaluated the cellular uptake and luciferase expression in Hep G2 cells, which ubiquitously express both the LDLR and ASGPR,^30^ to investigate the mechanisms by which the TriGalNAc-conjugated and unconjugated mRNA-LNPs interact directly with hepatocytes (Figure 3a). There was no clear enhancement in cellular uptake or luciferase expression with the addition of DSPE-PEG2000-TriGalNAc in LNPs 2, 18, and 20 compared to their unconjugated counterparts LNPs 1, 17, and 19 in the presence of serum (Figure 3b–d) or absence of serum (Figure 3e–g). While LNP 22, the composition that induced the highest luciferase expression in the liver *in vivo* in Figure 2, showed a slight enhancement in cellular uptake compared to the unconjugated counterpart (LNP 21) in the presence of serum (Figure 3b,c) or absence of serum (Figure 3e,f), luciferase expression was unchanged in both conditions (Figure 3d,g). Notably, LNP 22 showed the highest cellular uptake among DSPE-PEG LNPs 17–22 (Figure 3b,d,e,f). However, importantly, both the cellular uptake and luciferase expression levels induced by LNP 22 remained 5–11 folds lower than that of LNP 1 in the presence of serum (Figure 3b–d) and 3–5 lower in the absence serum (Figure 3e–g). LNP 1 and 2 also showed a much higher cellular uptake and luciferase expression levels compared to LNP 22 even when the mRNA dose was 4-fold lower (25 ng mRNA/well, Figure S6). Although the slightly improved cellular uptake of LNP 22 suggests that shortening the PEG Mw of the PEG-lipid stabilizer (e.g., DSPE-PEG350) could have facilitated LNP cellular uptake by better exposing the TriGalNAc ligands on the surface, the low overall in vitro cellular uptake and luciferase expression induced by LNP 22 relative to LNPs 1 did not correlate well with the in vivo delivery efficiency (Figure 2). This further emphasizes the weak in vitro/in vivo correlation of nucleic acid LNPs as described in the literature.^31^

**Figure 3:**
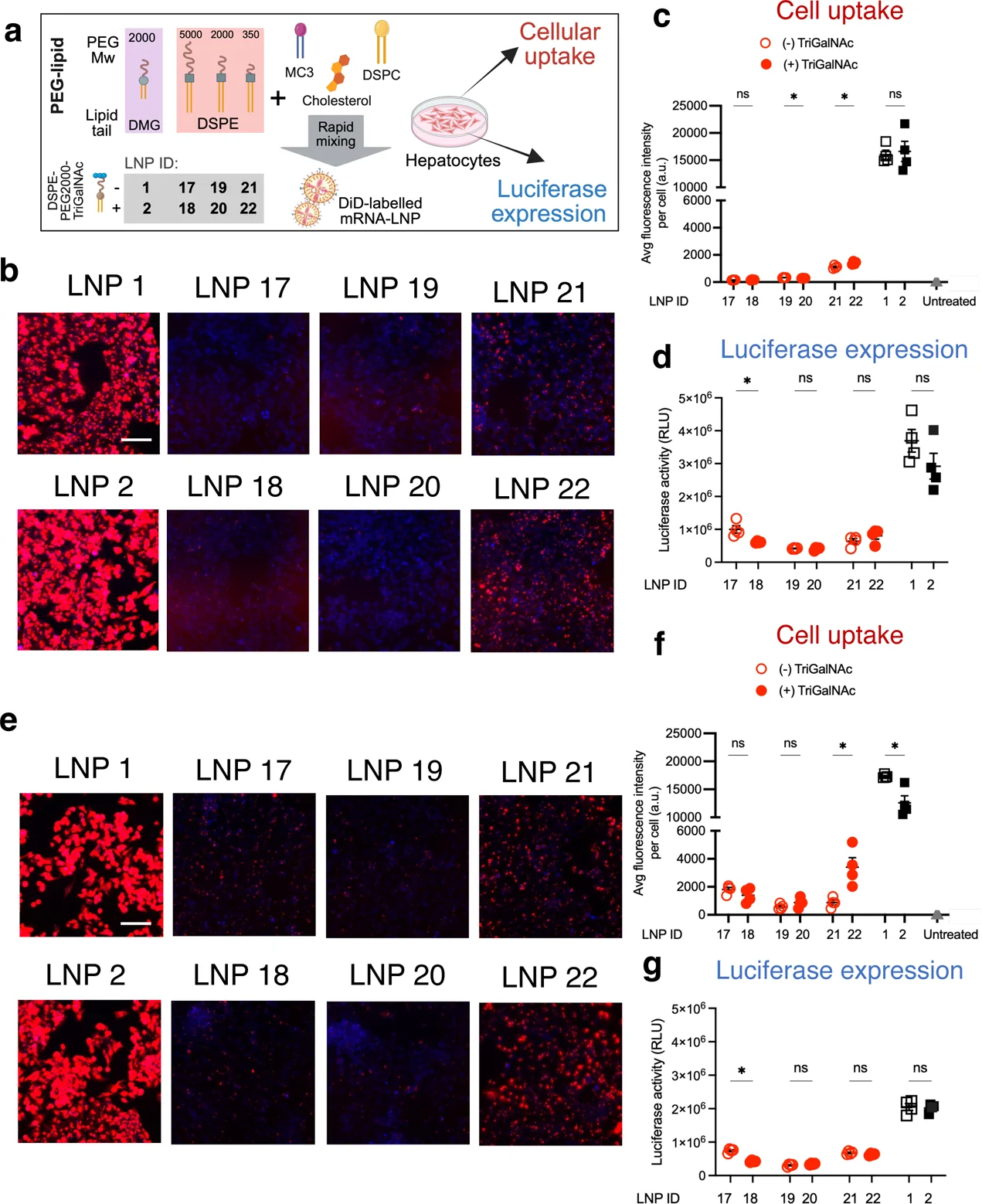
In vitro cellular uptake and luciferase expression analysis. (a) Illustration showing the different types of PEG-lipid stabilizers used to assess their impact on the cellular uptake and luciferase expression of TriGalNAc-conjugated and unconjugated mRNA-LNPs in Hep G2 cells. Labeled LNPs (0.1 mol % DiD) containing fLuc mRNA were added at 100 ng mRNA/well and further incubated for 24 h in in the presence of 10% fetal bovine serum (FBS) (b–d) or absence of FBS (e–g). (b,e) Representative fluorescent microscope images showing DiD uptake (red) and cell nuclei (blue). Scale bars represent 100 μm. (c,f) Quantification of the average DiD signal intensity per cell in each well. The data represent the mean ± SEM (*n* = 4 wells). (d,g) Quantification of luciferase expression in cell lysates after capturing the fluorescence images. The data represent the mean ± SEM (*n* = 4 wells). Statistical significance was calculated using two-tailed, unpaired *t* tests between the unconjugated and conjugated LNPs. ns: nonsignificant. *P < 0.05.

### 2.4 DSPE-PEG350 Moderately Increases LNP Circulation Time and Only Moderately Decreases mRNA Delivery Efficiency in Unconjugated LNPs

We next sought to investigate potential mechanisms by which LNP 22 outperformed Onpattro-like, DMG-PEG2000 LNPs in hepatic delivery efficiency in vivo. For a ligand-decorated nanoparticle to reach and bind to target receptors, nonspecific interactions with blood components must be minimized and elimination by the reticuloendothelial system (RES) avoided,^16,32^ even if the target cells are liver hepatocytes. The elimination half-life of nanoparticles following intravenous injection can serve as an indicator to assess the potential for their longevity in blood and subsequently increased exposure to target tissues and receptors.^33,34^ To evaluate how the PEG-lipid component influences the intrinsic pharmacokinetic profile of LNPs, we measured the circulation time of unconjugated, DiD-labeled mRNA-LNPs containing 1.5 mol % PEG-lipid following intravenous injection in mice (Figure 4a). Onpattro-like LNP (LNP 1) was rapidly cleared from the blood, with almost complete elimination within 1 h postinjection. DSPE-PEG2000 (LNP 24), excipient commonly used in long circulating liposomes, induced a longer circulation time and a 5.7-fold higher area under the curve (AUC) compared to LNP 1 (Figure 4b,c), which could be attributed to the anchoring effect of DSPE, slowing down PEG-lipid desorption and elongating LNP residence time in the blood.^8^ DSPE-PEG5000 LNP (LNP 25) showed the longest circulation time, which had an AUC 6.7-fold higher than that of LNP 1 (Figure 4b,c). Unexpectedly, DSPE-PEG350 (LNP 23) increased the AUC by 4-fold compared to LNP 1 and was only 1.4-fold lower than LNP 24, despite a 6-fold lower PEG Mw (350 g/mol vs 2000 g/mol) (Figure 4b,c).

**Figure 4:**
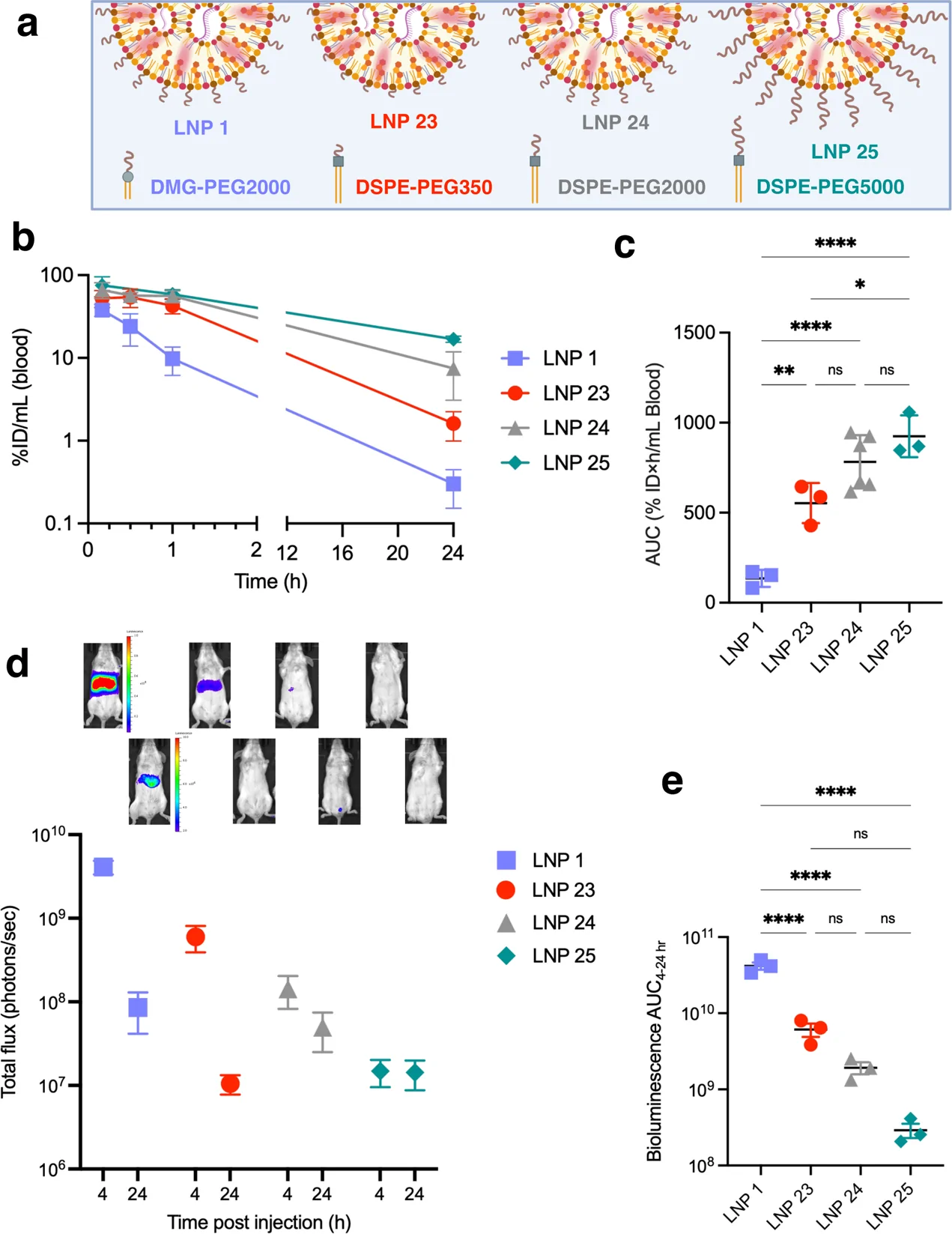
Unconjugated DSPE-PEG350 LNPs show moderately extended blood circulation without severely inhibiting luciferase expression in the liver. (a) Illustration showing the different types of PEG-lipids used to assess their impact on DiD-labeled LNP blood circulation and luciferase expression in the liver. LNPs were prepared using 1.5 mol % PEG-lipid, 0% DSPE-PEG2000-TriGalNAc, and 0.5 mol % DiD. (b) DiD-labeled mRNA-LNPs were intravenously injected into BALB/c mice. Blood samples were withdrawn periodically from the tail vein to quantify the amount of DiD remaining in the blood as a percentage of initial dose (ID)/ mL blood. The data represent the mean ± SD (*n* = 3 mice, except for LNP 23, *n* = 6). (c) Area under the curve (AUC) as calculated by the trapezoid rule. The data represent the mean ± SD (*n* = 3 mice, except for LNP 23, *n* = 6). Statistical significance was calculated using one-way ANOVA followed by Tukey’s posthoc analysis. ns: nonsignificant. *P < 0.05. **P < 0.01. ***P < 0.001. ****P < 0.0001. (d) Liver luciferase expression 4 and 24 h post intravenous injection of 7.5 μg fLuc mRNA in BALB/c mice, as quantified by whole-body imaging using IVIS. The data represent the mean ± SEM (*n* = 3 mice). (e) AUC of liver luciferase expression as calculated by the trapezoid rule. The data represent the mean ± SEM (*n* = 3 mice). Statistical significance was calculated using one-way ANOVA followed by Tukey’s posthoc analysis. ns: nonsignificant. *P < 0.05. **P < 0.01. ***P < 0.001. ****P < 0.0001. Representative IVIS images are shown. Images from all replicates are available in Figure S1.

Concomitantly, we measured the in vivo luciferase expression 4 and 24 h post intravenous injection of mRNA-LNPs to measure their intrinsic ability to deliver mRNA to the liver. DMG-PEG2000 (LNP 1) showed the highest luciferase expression in the liver (Figure 4d,e). LNPs containing DSPE-PEG2000 (LNP 24) or DSPE-PEG5000 (LNP 25) showed 20 and 60-fold reduction in luciferase expression levels in the liver compared to LNP 1, respectively (Figure 4e). LNP containing DSPE-PEG350 (LNP 23) was only 6-fold less efficient than LNP 1 in inducing luciferase expression in the liver (Figure 4e), but 3 and 21-fold more efficient than LNPs 24 and 25, respectively (Figure 4e). This indicates that while DSPE-PEG350 shows moderately increased circulation time (Figure 4b,c), there was also moderate reduction in hepatic luciferase expression relative to LNP 1 (Figure 4d,e).

To investigate the potential reasons behind the moderate reduction in the luciferase expression in the liver induced by unconjugated DSPE-PEG30 LNPs (e.g., LNP 23), we measured ApoE binding on the surface of LNPs after incubation with mouse plasma. Both LNPs 1 and 23 show comparable ApoE binding intensities (ementary Figure S7), suggesting that both LNPs are accessible for ApoE adsorption in the bloodstream after intravenous delivery. This subsequently suggests that while the binding of LNP 23 by the LDLR may still occur, the remaining, nonshedded DSPE-PEG350 on the LNP after adminstration could reduce LNP cellular uptake and the overall luciferase expression in the liver.^11^ We further confirmed that DiD-labeled LNP 23 show reduced cellular uptake of in Hep G2 cells, which was 6–17-fold lower than that of LNP 1 but 5–7-fold higher than that of LNPs 24 or 25 at 25 and 100 ng mRNA/well (Figure S8).

### 2.5 Incorporation of TriGalNAc-Lipid into DSPE-PEG350 LNPs Potentiates the Hepatic mRNA Delivery of Ionizable Cationic Lipids

We further studied the impact of pairing DSPE-PEG2000-TriGalNAc with DSPE-PEG350 in LNPs formulated with alternative ionizable cationic lipids (Figure 5a). As a proof of concept, we first replaced MC3 with an earlier-generation ionizable cationic lipid, DODAP (Figure 5a and Table S1). Similar to the observation with MC3 (Figures 1 and 2b–d), the addition of DSPE-PEG2000-TriGalNAc to DODAP LNPs containing DMG-PEG2000 did not improve luciferase expression in the liver (LNP 26 vs LNP 27, Figure 5b,c). However, the addition of DSPE-PEG2000-TriGalNAc to DODAP LNPs containing DSPE-PEG350 induced a 5.7-fold increase in luciferase expression in the extracted livers compared to the unconjugated LNP (LNP 29 vs LNP 28, Figure 5c). Additionally, at 4 h postinjection, LNP 29 induced 2-fold increase in luciferase expression in the liver compared to LNPs 26 or 27, further demonstrating the advantage of pairing DSPE-PEG350 with DSPE-PEG2000-TriGalNAc in boosting hepatic mRNA delivery (Figure 5b). However, the liver expression levels obtained by LNP 29 were 2 orders of magnitude lower than those obtained by LNP 22 (Figures 2k–m and 5b,c).

**Figure 5:**
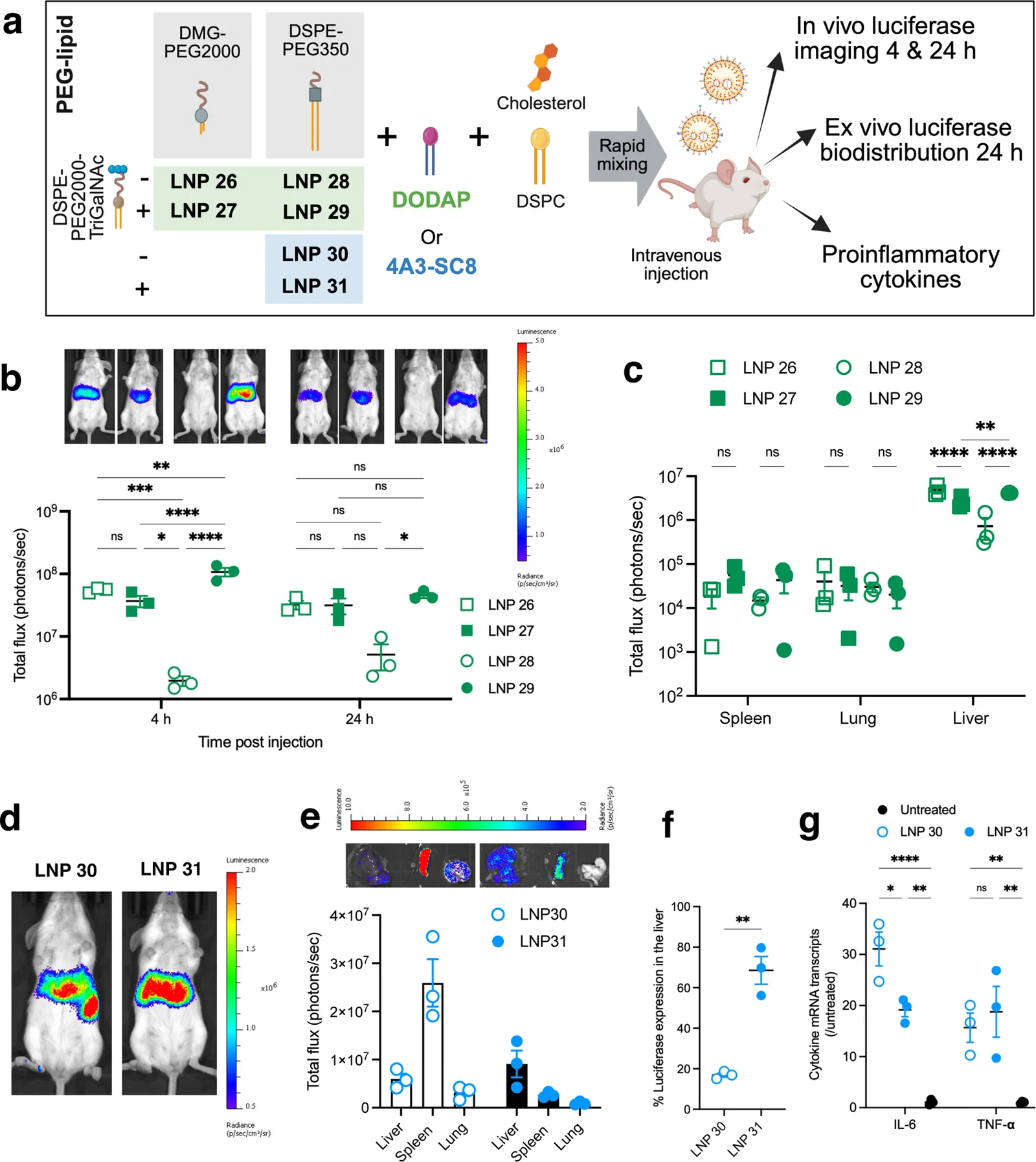
Pairing of DSPE-PEG350 with DSPE-PEG2000-TriGalNAc potentiates the hepatic mRNA delivery efficiency of ionizable cationic lipids. (a) Illustration showing the different types of PEG-lipids and ionizable cationic lipids used in formulating TriGalNAc-conjugated and unconjugated LNPs for intravenous administration. (b,c) DODAP LNPs. (d–g) 4A3-SC8 LNPs. (b) Liver luciferase expression 4 and 24 h post intravenous injection of 7.5 μg fLuc mRNA encapsulated in DODAP LNPs in BALB/c mice, as quantified by whole-body imaging using IVIS. (c) Ex vivo luciferase biodistribution in the extracted organs 24 h postinjection. LNPs were formulated using 1.5 mol % DMG-PEG2000 (LNPs 26 and 27) or 3 mol % DSPE-PEG350 (LNPs 28 and 29), with or without 0.1 mol % DSPE-PEG2000-TriGalNAc. The data represent the mean ± SEM (*n* = 3 mice). Statistical significance was calculated using two-way ANOVA followed by Tukey’s posthoc analysis. ns: nonsignificant. *P < 0.05. **P < 0.01. ***P < 0.001. ****P < 0.0001. (d) Whole-body imaging showing the liver and spleen luciferase expression of 4A3-SC8 LNPs coformulated with 3 mol % DSPE-PEG350 and DSPE-PEG2000-TriGalNAc at 0 mol % (LNP 30) or 0.1 mol % (LNP 31). LNPs were injected at 7.5 μg fLuc mRNA in BALB/c mice and imaged 4 h after (e) quantification of luciferase signal in extracted organs 4 h post injection (f) percentage of luciferase expression from the liver, calculated as a proportion of the total signal from liver, spleen, and lungs. (g) Quantification of TNF-α and IL-6 mRNA transcripts in the liver 4 h postinjection as quantified by RT-qPCR. The data represent the mean ± SEM (*n* = 3 mice). Statistical significance was calculated using one-way ANOVA followed by Tukey’s posthoc analysis. ns: nonsignificant. *P < 0.05. **P < 0.01. ***P < 0.001. ****P < 0.0001. Representative IVIS images are shown. Images from all replicates are available in Figure S1.

We then tested the hepatic delivery efficiency of 4A3-SC8 as an example of ionizable cationic lipid with low immunogenicity^35^ (Table S1). While 4A3-SC8 has been used to formulate various tissue-tropic LNPs,^36^ fully substituting the ionizable lipid with 4A3-SC8 in our DSPE-PEG350 LNPs produced large, polydisperse particles (LNP 30, Table S1). Consistent with previous reports demonstrating splenic accumulation of large, polydisperse mRNA lipoplexes or polyplexes,^37,38^ intravenous administration of LNP 30 resulted in high luciferase expression in the spleen. Interestingly, addition of 0.1 mol % DSPE-PEG2000-TriGalNAc (LNP 31) markedly reduced splenic luciferase expression and slightly increased its expression in the liver (Figure 5d,e). As a result, most of the total luciferase expression in the LNP 31-treated group originated from the liver (Figure 5f). To assess whether TriGalNAc functionalization altered the acute inflammatory response in the liver, we measured pro-inflammatory cytokine transcripts 4 h after intravenous administration of LNPs 30 and 31. TriGalNAc-conjugated LNP 31 did not increase IL-6 or TNF-α expression compared with unconjugated LNP 30 (Figure 5g), suggesting that TriGalNAc functionalization can be used to effectively restrict luciferase expression in the liver without exacerbating hepatic inflammation. However, the liver expression level obtained by LNP 31 was 3 orders of magnitude lower than those obtained LNP 22 at 4 h postinjection (Figures 2k and 5d,e). Note that luciferase expression at 24 h postinjection was not evaluated for 4A3-SC8 LNPs due to their low expression levels. Instead, the 4 h time point was shown in Figure 5d–f, as it represents the peak expression.

### 2.6 Physicochemical Characterization of the LNP Showing the Highest Hepatic mRNA Delivery Efficiency

We first plotted liver luciferase expression values at 4 h postintravenous injection for all LNPs formulated in this study (LNP 1-LNP 31; Table S1) to identify the most efficient liver-targeted system (Figure 6a). We identified 6 formulations (LNPs 6, 16, 2, 12, 10, and 22) that outperformed the Onpattro-like LNP (LNP 1) (Figure 6a). Although these 6 formulations contained a DSPE-PEG2000-TriGalNAc, the library also revealed numerous TriGalNAc-conjugated LNPs with lower delivery efficiency than LNP 1 (Figure 6a). This highlights the important role that nonligand LNP compositional elements play in potentiating the active targeting efficiency to the liver.

**Figure 6:**
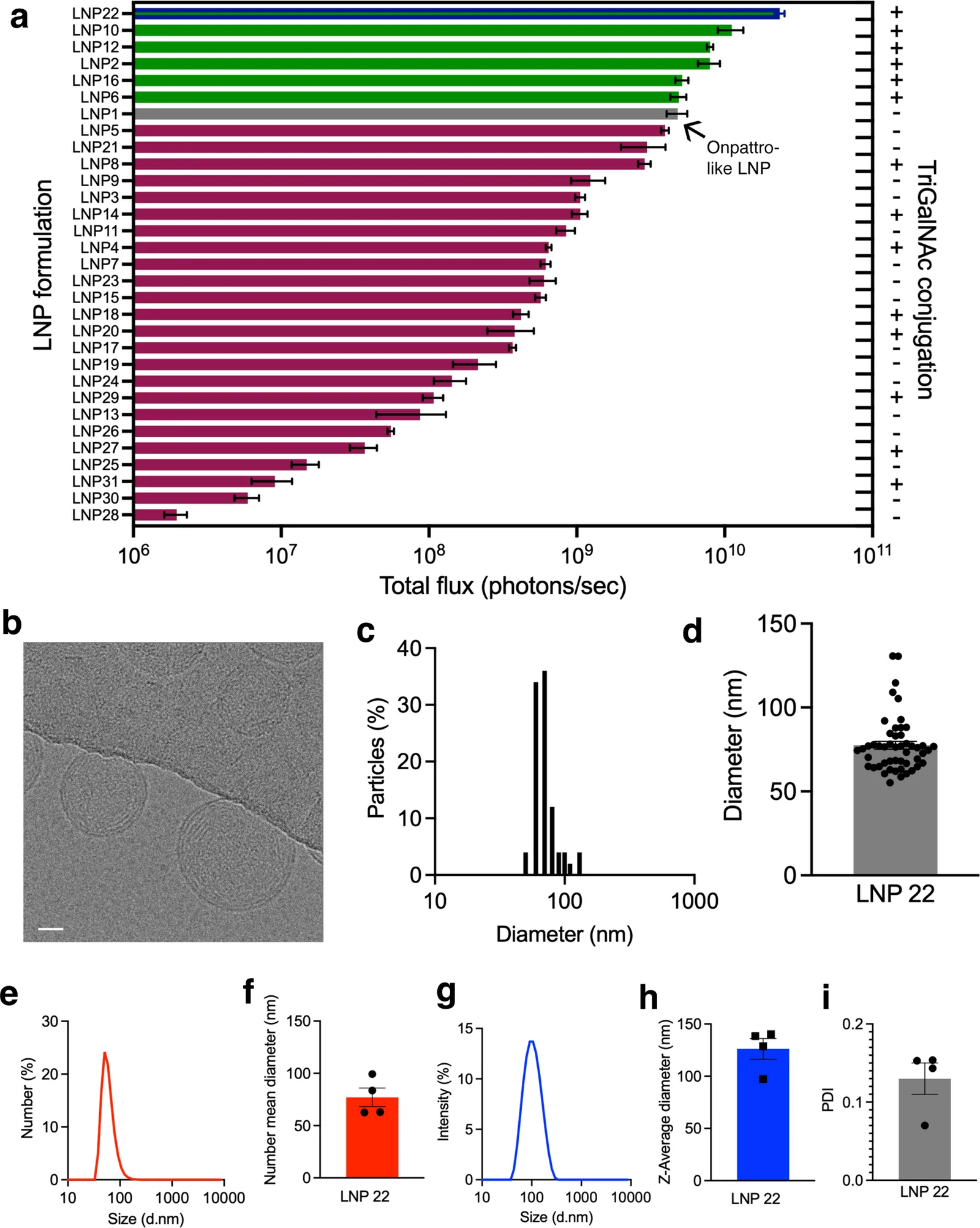
Identification of LNP 22 as the most efficient liver-targeted composition and its physicochemical characterization. (a) Luciferase expression in the liver of the mRNA-LNP library created in this study. The left *Y* axis denotes the LNP identification number (for composition details, refer to Table S1). The right *Y* axis indicates the presence or absence of 0.1 mol % DSPE-PEG2000-TriGalNAc in the LNP. The *X* axis represents the luciferase expression in the liver 4 h post intravenous injection of 7.5 μg fLuc mRNA in BALB/c mice, as quantified by IVIS. (b) Representative CryoTEM image of LNP 22. Scale bar = 20 nm. (c) Size-distribution histograms of LNP 22 as measured from CryoTEM images in Figure S9. *n* = 50 particles. (d) Average particle diameter of LNP 22 as estimated from the CryoTEM images (*n* = 50 particles). Individual particle diameters were calculated in ImageJ as the mean of the longest and shortest axes. (e) Representative size distribution histogram (number) of LNP 22 as measured by DLS. (f) Average number mean of 4 independent formulations. Mean ± SEM (g) Representative size distribution histogram (intensity) of LNP 22 as measured by DLS. (h) Average *Z*-average size of 4 independent formulations. Mean ± SEM (i) Average PDI of 4 independent formulations as measured by DLS. Mean ± SEM.

To observe the effect that pairing of DSPE-PEG350 and DSPE-PEG2000-TriGalNAc have on LNP morphology or mRNA conformation within the core, both of which could affect the delivery efficiency,^39,40^ we performed cryogenic transmission electron microscopy (cryoTEM) imaging. LNP 22 exhibited a spherical/ellipsoid structure with no detectable mRNA blebs, as shown in the representative image obtained at high magnification (Figure 6b), and an extended panel of images obtained at lower magnification (Figure S9). Further, average particle diameter calculated from cryoTEM was comparable to the number mean diameter obtained by dynamic laser scattering (DLS), which was around 77 nm (Figure 6c–f). The *Z*-average size and polydispersity index (PDI) derived from DLS measurements were around 126 nm and 0.13, respectively (Figure 6g–i), indicating the low heterogeneity of particle distribution.

We also checked the anti-PEG antibody production induced by LNP 22 in mice, because excessive production of anti-PEG antibodies could limit repeated administration (Figure S10). While TriGalNAc-conjugated LNPs based on DSPE-PEG5000 (LNP 18) and DSPE-PEG2000 (LNP 20) showed a relatively high production of anti-PEG IgM in mouse plasma 1 week after a single intravenous injection, TriGalNAc-conjugated LNP based on DSPE-PEG350 (LNP 22) showed lower IgM production, which was comparable to the levels included by TriGalNAc-conjugated LNP based on DMG-PEG2000 (LNP 6) (Figure S10). Note that all formulations in Figure S10 contained 0.1 mol % DSPE-PEG2000-TriGalNAc and 3 mol % PEG-lipid to unify the PEG content for direct comparison, as small differences in PEG content may affect the induction of anti-PEG antibodies.

### 2.7 Incorporation of DSPE-PEG2000-TriGalNAc into DSPE-PEG350 LNPs Results in Improved Hepatic Base Editing

Lastly, we sought to assess whether the improved delivery efficiency of LNP 22 could facilitate more potent RNA therapeutics. Base editors can mediate efficient in vivo single nucleotide modifications that durably treat genetic disease in mouse models and are currently in clinical trials.^41^ To evaluate whether TriGalNAc incorporation in LNPs can improve base editing efficiency, we selected LNPs 1 and 22 as delivery vehicles to test in mice. First, we coencapsulated ABE8e mRNA and a single guide RNA (sgRNA) (2:1, wt. ratio) in LNPs (Table S1). The sgRNA (sgDNMT1) is targeted to an established test site in the DNMT1 gene in mice.^42^ Seven days after intravenous injection of 20 μg total RNA in wild-type mice, mice treated with LNP 22 (DSPE-PEG350 paired with DSPE-PEG2000-TriGalNAc) induced a 25.6% base conversion rate in the liver, which was 2.7-fold higher than the editing observed after treatment with the same dose of LNP 1 (Onpattro-like LNP) (Figure 7a,b). We also did not observe any significant base editing in extrahepatic tissues (Figure 7c). Although there was a statistical significance between the untreated group and LNP 22-treated group in the spleen, the base A-G conversion rate remained low, not exceeding 0.6% (Figure 7c).

**Figure 7:**
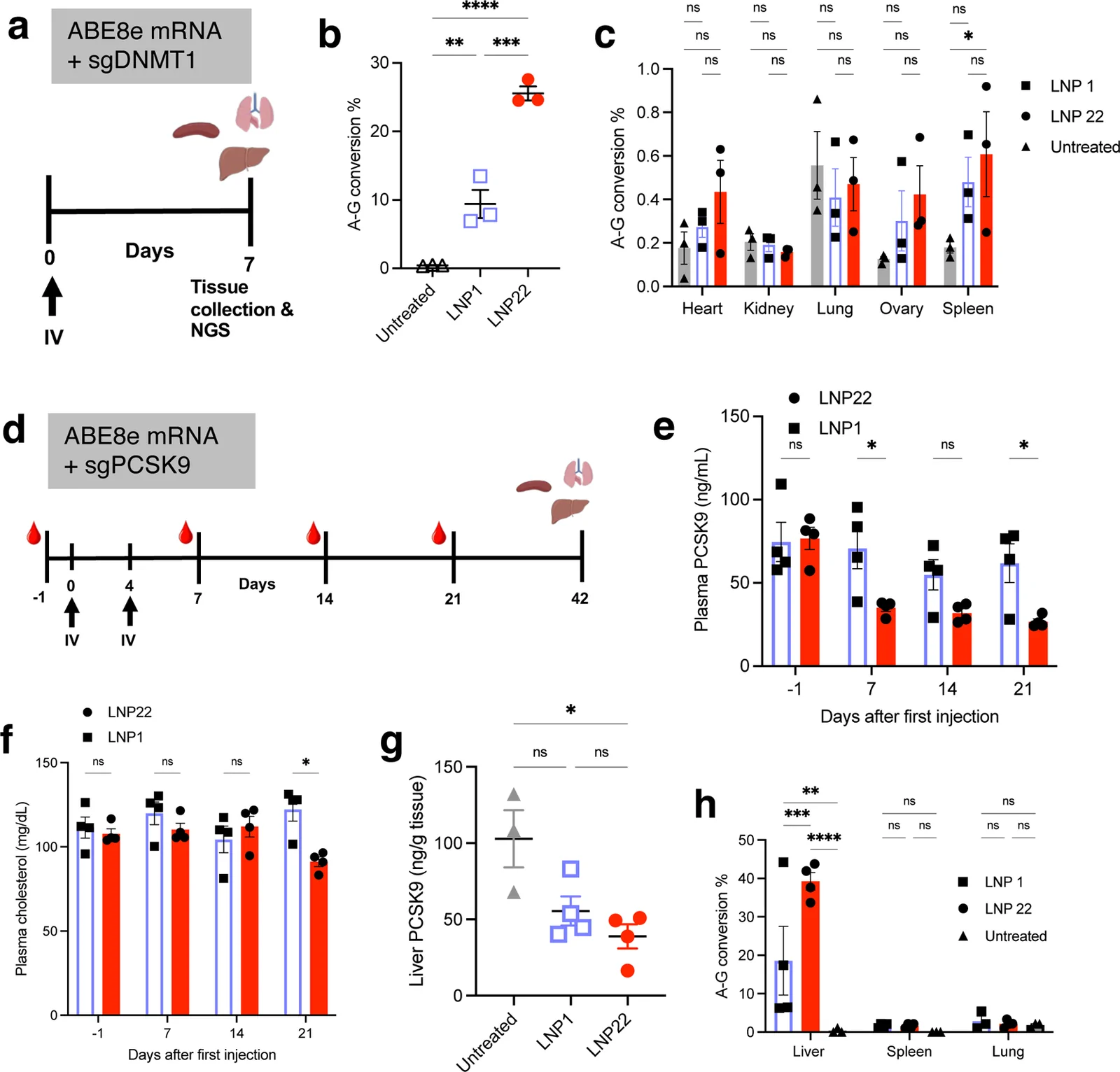
Pairing of DSPE-PEG350 with DSPE-PEG2000-TriGalNAc enables efficient hepatic base editing. (a) Treatment schedule of LNPs coencapsulating ABE8e mRNA and sgDNMT1 (2:1) in BALB/c mice. Mice were intravenously injected with 20 μg total RNA either in LNP 1 (1.5 mol % DMG-PEG2000, 0 mol % DSPE-PEG2000-TriGalNAc) or LNP 22 (3 mol % DSPE-PEG350, 0.1 mol % DSPE-PEG2000-TriGalNAc). (b) Livers were analyzed for base conversion rate 7 days postinjection by NGS. The data represent the mean ± SEM (*n* = 3 mice). One-way ANOVA followed by Tukey’s posthoc analysis. **P < 0.01. ***P < 0.001. ****P < 0.0001. (c) Off-target base editing in extrahepatic tissues. The data represent the mean ± SEM (*n* = 3 mice). Two-way ANOVA followed by Tukey’s posthoc analysis. ns: nonsignificant. *P < 0.05. (d) Treatment schedule of LNPs coencapsulating ABE8e mRNA and sgPCSK9 (2:1) in BALB/c mice. Mice received 2 injections with 20 μg total RNA either in LNP 1 (1.5 mol % DMG-PEG2000, 0 mol % DSPE-PEG2000-TriGalNAc) or LNP 22 (3 mol % DSPE-PEG350, 0.1 mol % DSPE-PEG2000-TriGalNAc). (e) Plasma PCSK9 protein levels as measured by ELISA. The data represent the mean ± SEM (*n* = 4 mice). Two-tailed, unpaired *t* tests. ns: nonsignificant. *P < 0.05. (f) Plasma cholesterol levels as measured by the colorimetric assay kit. The data represent the mean ± SEM (*n* = 4 mice). Two-tailed, unpaired *t* tests. ns: nonsignificant. *P < 0.05. (g) Quantification of liver PCSK9 protein levels 5 weeks after the first intravenous injection, as measured by ELISA in liver homogenates. The data represent the mean ± SEM (*n* = 4 mice). One-way ANOVA followed by Tukey’s posthoc analysis. ns: nonsignificant. *P < 0.05. (h) Quantification of base conversion rate by NGS. The data represent the mean ± SEM (*n* = 4). Two-way ANOVA followed by Tukey’s posthoc analysis. ns: nonsignificant. *P < 0.05. **P < 0.01. ***P < 0.001. ****P < 0.0001.

We also delivered ABE8e-endocing mRNA and sgPCSK9 to disrupt the splice donor at the boundary of Pcsk9 exon 1 and intron 1, as previously established.^43,44^ PCSK9 is a key therapeutic factor for the management of hypercholesterolemia and cardiovascular disease that is primarily synthesized and secreted by hepatocytes.^45,46^ Two intravenous injections of LNP 1 or LNP 22, each delivering 20 μg total RNA (ABE8e mRNA: sgPCSK9, 2:1), were administered to wild-type mice (Figure 7d). While LNP 1 reduced circulating PCSK9 protein levels in mouse plasma only by 17% by day 21 after initiating the treatment, LNP 22 lowered plasma PCSK9 protein levels by 65% (Figure 7e). Further, LNP 22 induced a statistically significant reduction in plasma cholesterol levels in wild-type mice fed normal diet at day 21 of initiating treatment compared to LNP 1 (Figure 7f). Additionally, we measured PCSK9 protein levels and base editing efficiency in the liver 42 days after the first injection to assess the durability of base editing mediated by the LNPs (Figure 7g). Only LNP 22 showed a statistically significant reduction in PCSK9 protein levels in the livers compared to the untreated control, which was around a 60% reduction (Figure 7g). Finally, LNP 22 mediated 40% PCSK9 editing efficiency in the liver, which was 2-fold higher than that of LNP 1 (Figure 7h). A previous report showed that traditional mRNA-LNP did not induce measurable base editing at doses as low as 1 mg/kg per animal.^47^ We did not observe any significant base editing in extrahepatic tissues, including the lung and spleen, with LNP-treated mice (Figure 7h).

## 3 Discussion

Ionizable LNPs exhibit a unique hepatic delivery mechanism, wherein protein corona formation and PEG-lipid desorption direct LNP uptake by the liver, primarily by the LDLR. As a result, LNPs are short-circulating and rapidly cleared from the blood compartment soon after intravenous administration.^48^ Our data show that incorporating TriGalNAc ligands into short-circulating mRNA-LNPs based on DMG-PEG2000 did not further improve hepatic mRNA delivery compared to the unconjugated LNP. This was consistent with a previous study showing that the incorporation of a TriGalNAc-lipid into ionizable LNPs does not further enhance their hepatic base editing efficiency over unconjugated LNPs in wild-type mice or nonhuman primates but can induce efficient base editing efficiency in transgenic mice with reduced LDLR expression, where ionizable LNP uptake is reduced.^21^ In addition, a previous study assessing the impact of TriGalNAc modifications on the hepatic delivery of small interfering RNA (siRNA) showed that the incorporation of TriGalNAc-lipid into DMG-PEG ionizable LNPs did not improve gene silencing compared to unconjugated LNPs in wild-type mice.^48^ While shielding the LNP with a dense, stable coating of PEG-lipid before adding the TriGalNAc-lipid achieved better specificity to the ASGPR uptake pathway, the overall hepatocyte gene silencing efficiency induced by TriGalNAc-LNPs in wild-type or LDLR knockout mice was weaker than that induced by the unconjugated, ionizable LNPs in wild-type mice.^48^ It remains unclear whether active targeting using TriGalNAc ligands can outperform the delivery efficiency induced by canonical ionizable LNPs in wild-type animals. Achieving this could improve the editing efficiency of mRNA-LNPs and expand their applicability to a wider range of genetic disorders, regardless of whether the hepatic LDLR expression is normal or reduced.^20^

Stealth nanomedicines, which reduce nonspecific interactions with blood components and clearance by the RES, can potentially serve as platforms for ligand-mediated active targeting to cancer cells, hepatocytes, or the brain endothelium and parenchyma.^33,34,49^ Traditionally, PEGylation is used to confer stealth properties in lipid-based nanoparticles, either by increasing the PEG concentration, PEG Mw, or PEG-lipid tail Mw for better anchoring in the lipid bilayer.^34^ While PEGylation may reduce blood clearance and enhance mRNA accumulation in target tissues, it can interfere with postdelivery processes, including cellular uptake and endosomal escape in target cells.^9,27^ Indeed, our data show that some LNPs coformulated with PEG-lipids bearing a high Mw PEG (e.g., 2000–5000 g/mol) or long lipid tail (e.g., DSPE) provided low luciferase expression in the liver and cellular uptake in vitro that was not further improved with the addition of DSPE-PEG2000-TriGalNAc. Therefore, a strategy to prolong circulation time while preserving intrinsic LNP uptake capacity could lead to enhanced TriGalNAc-mediated mRNA delivery to hepatocytes.

Several alternative approaches have emerged to increase the longevity of LNPs in the blood by modifying the lipid composition or using RES uptake-blocking strategies.^50–52^ Here, we focused on the effect of PEG-lipid structure with and without pairing with TriGalNAc ligands on (i) the in vivo hepatic luciferase expression, (ii) in vitro cellular uptake, and (iii) circulation properties of mRNA-LNPs using select formulations. We discovered that substituting DMG-PEG2000 or DSPE-PEG2000, PEG-lipids used in FDA-approved lipid-based products, with DSPE-PEG350 and pairing it with DSPE-PEG2000-TriGalNAc (LNP 22) improved hepatic luciferase expression by 5–60-fold in wild-type mice. Surprisingly, we found that unconjugated DSPE-PEG350 LNP exhibited moderately extended circulation time following intravenous injection, exceeding that of DMG-PEG2000 and comparable to that of DSPE-PEG2000. At the tissue level, unconjugated DSPE-PEG350 LNPshowed reduced accumulation in the liver, but increased accumulation in other organs such as the lung and spleen, presumably due to increased circulation half-life and tissue exposure. While the mechanisms by which a relatively short PEG (DSPE-PEG350) can lengthen blood circulation remains to unclear, a recent study suggests that PEG-lipids may enhance the stealth properties of liposomes not only through classical steric stabilization, but also by dehydrating the surface and promoting interzwitterionic ion pairing, offering a steric repulsion-independent mode of stabilization.^53^ In addition, we also found that unconjugated DSPE-PEG350 LNPs still retained ApoE biding affinity in vitro and did not completely inhibit cellular uptake and luciferase expression in the liver. DSPE-PEG350 may permit ApoE adsorption due to moderate stealth properties and are less likely to obstruct cellular uptake or endosomal escape compared to DSPE-PEG2000 and DSPE-PEG5000. Taken together, DSPE-PEG350 may contribute to an improved balance between the pharmacokinetic and cell uptake properties needed to boost active targeting after the addition of hepatocyte-targeting ligands (i.e., LNP 22). However, the full characterization of protein corona formation of LNP 22 in vivo and its role on ligand exposure and uptake by each of the LDLR or ASGPR pathways remains to be studied in the future using advanced analytical methods and transgenic mouse models.

From an avidity viewpoint, it is possible that the incorporation of a short stabilizer PEG could help extend the spacer PEG in a brush configuration on the surface of LNPs by steric repulsion,^54,55^ thus, enhancing ligand exposure and receptor interactions. This could partially explain the enhanced delivery efficiency observed in not only LNP 22, but also LNPs containing a PEG-lipid bearing a low Mw PEG (350–500 g/mol). However, our in vitro studies showed that LNP 22 induced only a modest improvement in cellular uptake compared to the unconjugated counterpart (LNP 21), which remained several folds lower than that of the Onpattro-like LNP (LNP 1). Although a previous report showed that a PEG350 surface coating enhanced the in vitro cellular uptake of targeting peptide-decorated liposomes, pairing the stabilizer PEG coating with a short PEG ligand spacer (ethylene glycol 12; ∼PEG540) was necessary, as liposomes with a PEG2000 ligand spacer and PEG350 surface coating showed diminished cellular uptake.^56^ This was opposite to our findings in vivo, showing that the pairing of DSPE-PEG350 and DSPE-PEG2000-TriGalNAc induced the highest in vivo hepatic mRNA delivery efficiency among all tested surface compositions in this study. Collectively, these findings underscore the importance of engineering the LNP surface structure to balance pharmacokinetics, ligand-mediated targeting, and cellular uptake in vivo, which may not be accurately predicted by *in vitro* studies only. It is noteworthy that the relatively small molecular weight of the ligand (i.e., TriGalNAc) in this study may have contributed to the ease of observing the effects arising from subtle changes in PEG-lipid structure. Whether similar outcomes would be achieved with larger targeting moieties, such as antibodies, remains to be determined.

We also observed that PEG-lipids containing DMPE-PEG2000 and DSG-PEG2000 (not DMG-PEG2000 or DSPE-PEG2000) paired with DSPE-PEG2000-TriGalNAc showed significant enhancement in hepatic mRNA delivery compared to the unconjugated counterparts. This could indicate that there is an optimal balance in the hydrophobicity of the PEG2000-lipid that may be crucial to potentiate the active targeting efficiency when paired with DSPE-PEG2000-TriGalNAc. Lower Mw lipid tails (e.g., DMG-PEG2000) may shed too readily, leading to shortened circulation times and insufficient active targeting efficiency. In contrast, higher Mw lipid tails (e.g., DSPE-PEG2000) may reduce cellular uptake and endosomal escape and ultimately limiting the overall active targeting efficiency. Intermediate-Mw lipid tails (e.g., DMPE-PEG2000 and DSG-PEG2000) may provide a more favorable balance of PEG-lipid shedding rates and induce an enhancement in overall active targeting efficiency. Although a full characterization of PEG-lipids shedding rates and their impact on cellular uptake is beyond the scope of the present study, it remains an important direction for future work.

While ionizable cationic lipids have been instrumental to the success of LNP-mediated delivery of nucleic acid therapeutics, they also introduce several limitations, including immunogenicity and storage instability.^7,13,57^ Therefore, enabling hepatic mRNA delivery through ligand-mediated targeting could offer greater flexibility in ionizable lipid selection and allow dose reduction. DODAP, a first-generation ionizable cationic lipid, is characterized by low mRNA delivery efficiency, presumably due to its low acid dissociation constant (p*K*_a_).^58^ Here, we achieved a 2-fold increase in liver luciferase expression induced by DODAP through the substitution of DMG-PEG2000 with DSPE-PEG350 and then pairing it with DSPE-PEG2000-TriGalNAc. We also show that our pairing strategy could potentiate mRNA delivery efficiency in the liver by reducing off-target expression, as seen with the 4A3-SC8 LNPs formulated with DSPE-PEG350 and DSPE-PEG2000-TriGalNAc, without aggravating the inflammatory response in the liver.

LNP 22 showed a morphology that is consistent with prior observations for DMG-PEG2000 LNPs characterized with a membrane-rich internal structure, which showed the highest stability and mRNA delivery efficiency among other tested LNP polymorphic shapes.^40^ Finally, we showcased the efficacy of LNP 22 in base editing of two gene targets in wild-type mice, DNMT1 and PCSK9, at low doses as low as 1 mg/kg of RNA per mouse. Dose reduction is important both to prevent toxicity as well as to reduce the cost of manufacturing genome editing LNPs. Additionally, LNP 22 could be more suitable for repeated administration, as it triggered low levels anti-PEG antibody, comparable to those seen with the sheddable DMG-PEG2000.^59^ This could be attributed to relatively low PEG Mw in DSPE-PEG350, which is lower that the antigen epitope size of ∼700 Da, as estimated in previous studies.^60,61^

## 4 Conclusion

We characterized a novel strategy to enhance the active targeting of ligand-conjugated mRNA-LNPs by controlling the selection of stabilizer and ligand spacer PEG-lipids. We discovered that incorporating DSPE-PEG2000-TriGalNAc-lipid into LNPs containing DSPE-PEG350 substantially enhanced hepatic mRNA delivery, outperforming TriGalNAc-conjugated or unconjugated LNPs that contain clinically used excipients such as DMG-PEG2000 or DSPE-PEG2000. To our knowledge, this is the first report demonstrating the synergistic enhancement of hepatocyte mRNA delivery enabled by incorporating targeting ligands onto ionizable LNPs, with potentially wider applicability in treating various liver diseases.

## 5 Materials and Methods

### 5.1 Materials

CleanCap AG Firefly luciferase and CleanCap M6 mCherry mRNA (N1MePsU) were purchased from Trilink Biotechnologies (San Diego, CA, USA). EZ Cap Cy5 Firefly Luciferase mRNA (5-moUTP) was purchased from APExBIO. Distearoylphosphatidylcholine (DSPC), DSPE-PEG2000, DSPE-PEG5000, DMG-PEG2000, DSPE-PEG350, Dimyristoylphosphatidylethanolamine (DMPE)-PEG350, DMPE-PEG2000, DSPE-PEG2000-TriGalNAc, and (Dioleoyl dimethylammonium propane) DODAP were purchased from Avanti Research. Distearoyl glycerol (DSG)-PEG500 and DMG-PEG500 were purchased from PurePEG, LLC. D-Lin-MC3-DMA (MC3), 4A3-SC8, DMG-PEG5000, and Tri-GalNAc-DBCO were purchased from MedChemExpress. DSPE-PEG8-Azide and DSG-PEG2000 were purchased from BroadPharma. Cholesterol was obtained from Sigma. DiD′ solid (1,1′-dioctadecyl-3,3,3′,3′-tetramethylindodicarbocyanine, 4-chlorobenzenesulfonate salt) was purchased from Invitrogen. D-luciferin and the mouse PCSK9 ELISA kit were purchased from Abcam. US Origin Mouse CD1 Plasma was obtained from Innovative grade Inc. The cholesterol E kit was purchased from FujiFilm. Mouse Anti-PEG IgM ELISA was obtained from Life Diagnostics. PerCP/Cyanine5.5 antimouse CD45 Antibody, FITC antimouse CD31 (PECAM-1) Antibody, and the Zombie Violet Fixable Viability Kit were obtained from Biolegend.

### 5.2 LNP Formulation and Characterization

LNPs were formulated by a rapid mixing method as previously described.^62^ A 200 μL ethanolic solution containing ionizable lipid, DSPC, Cholesterol, PEG-lipid, and TriGalNAc-conjugated lipid was prepared and mixed with 25 μL of 1 mg/mL mRNA dissolved in 1 mM sodium citrate pH 6.4. The nitrogen to phosphate (N/P) ratio of the nitrogen groups in the ionizable cationic lipid and the phosphate groups in the mRNA was set at 6. The mixture was then added dropwise to 600 μL of 50 mM sodium citrate buffer (pH 3) and let to equilibrate for 5 min at room temperature. The resulting mixture was then diluted in 30 mL PBS and spun using 30kD Amicon centrifugal filters (Millipore, MA, USA) for buffer exchange and removal of residual ethanol. The final formulation volume was brought to 250 μL and used for in vivo experiments or physicochemical characterizations. The TriGalNAc-lipid concentration was fixed at 0.1 mol % of total lipids because a previous study showed that the maximum delivery efficiency of TriGalNAc-lipid-modified mRNA-LNPs occurs at 0.05–0.25 mol % in *ldlr* ^–/–^ mice.^21^ For gene editing experiments, a 2:1 wt/wt ratio of ABE8e and sgRNA was premixed to formulate the LNPs. For labeling with a fluorescent dye, DiD was added to the lipid phase at a final concentration of 0.5 mol % in blood circulation measurements, and 0.1 mol % *in vitro* cell culture studies. Particle size and the polydispersity index (PDI) were measured using dynamic light scattering (DLS) equipped with a diode laser (λ = 532 nm) with a scattering angle of 173° (Zetasizer Nano-ZS, Malvern Instruments, Worcestershire, UK) at 10× dilution in PBS. To prepare DSPE-PEG350-TriGalNAc, 0.25 mM DSPE-PEG8-Azide was mixed with Tri-GalNAc-DBCO in DMSO at a 1:3 molar ratio. The reaction was allowed to take place for 24 h under continuous stirring at room temperature. The product was added in the lipid phase before mixing to make LNPs at 0.1 mol % of the initial DSPE-PEG8-Azide concentration. Unconjugated TriGalNAc-DBCO was removed by filtration during the buffer exchange step of LNP preparation.

### 5.3 Animals

All animal experiments were performed following the protocols evaluated and approved by the Johns Hopkins University Animal Care and Use Committee (Protocol Approval Number: MO24M312). Johns Hopkins Program of Animal Care and Use is accredited by AAALAC International. Animal care and procedures follow the Guide for the Care and Use of Laboratory Animals eighth edition.^63^ Female BALB/c mice (7 weeks) were purchased from Charles River Laboratories and were housed in a 12 light/12 dark cycle room. Mice were given normal fat diet.

### 5.4 Quantification of Luciferase Expression In Vivo

Mice were administered LNPs at a dose of 7.5 μg mRNA per mouse by tail vein injections. Luciferase expression from living animals was quantified at 4 and 24 postinjections using an in vivo imaging system (IVIS, PerkinElmer). Images were acquired 10 min after i.p. injection of 200 μL of 15 mg/mL luciferin substrate and 10 s exposure time. The total flux of luminescence was calculated by gating a region of interest in the upper abdomen area. For quantifying luciferase activity in mouse organs, liver, lung, and spleen were extracted immediately after sacrifice and imaged using IVIS. For quantifying Cy5 fluorescence intensity, mice were sacrificed and organs were imaged at excitation λ = 620 nm and emission λ = 670 with 10 s exposure time.

### 5.5 In Vitro Cell Culture Studies

Hep G2 cells were seeded at 8000 cells per well in 40 μL of culture media (DMEM containing 10% fetal bovine serum (FBS) and 1% Penicillin–Streptomycin) in a 384-well plate at 37 °C and 5% CO_2_. One day after seeding, media was removed and the DiD-labeled LNPs containing fLuc mRNA were dispersed in DMEM only (serum −) or DMEM containing 10% FBS (serum +) and added at 2 different concentrations: 25 and 100 ng mRNA per well. The cells were further incubated for 24 h, washed 3× with PBS, and the nuclei were stained with Hoechst (Thermo Fisher). Images were captured with the 20× objective in 16 fields per well using the high content imager (CellInsight CX7 LZR Pro, Thermo Fisher). Images were analyzed with a custom algorithm created using the HCS Studio Target-Activation image-analysis software package (Thermo Fisher), as previously described.^64^ With this algorithm, we use the nuclear stain to identify cells then quantify the DiD-based fluorescence intensity per cell and reported as average intensity/cell on a well-by-well basis. A representative field image from one well of each treatment group was shown, and the average DiD fluorescence intensity/cell in each well was presented as the mean ± SEM (*n* = 4). After imaging, cells were lysed with Bright-Glo (Promega) for 5 min and luminescence measured using the high-content luminometer (CLARIOstar, BMG Labtech), and presented as the mean ± SEM (*n* = 4).

### 5.6 Blood Circulation Measurements

Mice were administered DiD-labeled LNPs at a dose of 10 μg mRNA per animal by tail vein injection. At definite time points postinjection, 10 μL blood was withdrawn from the tail vein using heparinized tips and dissolved immediately in 230 μL 1% w/v sodium lauryl sulfate. A standard curve was prepared for each LNP using blood from untreated mice. Fluorescence intensity was determined using a Tecan plate reader at emission and excitation wavelengths 624 and 669 nm, respectively.

### 5.7 Quantification of Proinflammatory Cytokine mRNA in the Liver

Livers were extracted 4 h after LNP administration and stored at −80 °C until processing. Livers were homogenized in 1.5 mL TRIzol reagent. Following homogenization, 300 μL of chloroform was added to TRIzol tissue homogenate. Tubes were spun down, and the supernatants were collected. The supernatant was processed with RNAeasy kit (Qiagen) to yield purified RNA. RNA concentration and quality were measured using an ND-2000 nanodrop (Thermo Fisher Scientific). Complementary DNA was produced using a high-capacity cDNA reverse transcription kit (Thermo Fisher Scientific). Quantitative Polymerase chain reaction (qPCR) was performed on QuantStudio 3. Levels of IL6, TNFα, and reference gene RPLP0 were measured using the following primers: IL6-F: TACCACTTCACAAGTCGGAGGC; IL6-R: CTGCAAGTGCATCATCGTTGTTC; TNFα-F: GGTGCCTATGTCTCAGCCTCTT; TNFα-R: GCCATAGAACTGATGAGAGGGAG; RPLP0-F: GCTTCGTGTTCACCAAGGAGGA; RPLP0-R: GTCCTAGACCAGTGTTCTGAGC. Results were analyzed using the ΔΔCt method.

### 5.8 Cryogenic Transmission Electron Microscope Imaging

fLuc mRNA-loaded LNP was prepared as described in Section 5.2 and subsequently concentrated 5-fold. A 3 μL aliquot of the mRNA-LNP was applied to a Quantifoil R1.2/1.3, 300-mesh grid. The grids were plunge-frozen using a Vitrobot IV (Thermo Fisher Scientific). Cryo-TEM imaging was performed on a Talos Glacios (Thermo Fisher Scientific) operated at 200 kV and equipped with a Falcon 4i detector. Particles with clearly discernible boundaries were analyzed by measuring the longest and shortest diameters of each particle in ImageJ, and the average of these two measurements was used as the representative LNP size.

### 5.9 In Vitro Transcription of ABE8e mRNA

ABE8e PCR product was amplified using the following forward primer that corrects the inactive T7 promoter on the plasmid, preventing round-the-plasmid transcription: 5′-TCGAGCTCGGTACCTAATACGACTCACTATAAGG-3′. The following reverse primer was used to add a 120nt polyA tail: 5′-TTTTTT­TTTTTT­TTTTTT­TTTTTT­TTTTTT­TTTTTT­TTTTTT­TTTTTT­TTTTTT­TTTTTT­TTTTTT­TTTTTT­TTTTTT­TTTTTT­TTTTTT­TTTTTT­TTTTTT­TTTTTT­TTTTTT­TTTTTC­TTCCTA­CTCAGG­CTTTAT­TCAAAG­ACCA-3′. PCR consisted of a 2 min denaturation at 98 °C followed by 30 cycles of (15 s denaturation at 98 °C, 20 s annealing at 71.4 °C, and 1.5 min extension at 72 °C), followed by a final extension at 72 °C for an additional 2 min. Amplified DNA was purified using a Qiagen PCR cleanup kit and quantified by nanodrop. To transcribe RNA, we used the NEB T7 HiScribe kit (Cat. No. E2040L). Transcription was conducted according to manufacturer instructions, using full substitution of UTP for N1-methylpseudouridine-5′-triphosphate (Trilink Cat. No. N-1081) and with 100 mM CleanCap AG (Trilink Cat. No. N-7113) and with 1 μg of DNA per 40 μL transcription reaction. Transcription was conducted for 2 h at 37 °C. Following transcription, mRNA was purified using lithium chloride precipitation. 7.5 M lithium chloride (Thermo Fisher Scientific Cat. No. AM9480) was diluted to a final concentration of 2.5 M directly in the transcription solution. The solution was chilled at −20 °C for 30 min. Precipitated mRNA was pelleted by centrifuging samples at 12,000*g* on a benchtop microcentrifuge for 15 min. The supernatant was discarded, and the pellet was washed with chilled 70% ethanol using a slightly larger volume than the original supernatant. After inversion and vortexing, mRNA was pelleted again by a second centrifugation at 12,000*g* for 15 min. Supernatant was discarded and residual ethanol was pipetted away and dried by placing open tubes in a biosafety cabinet to dry for 2 min. All mRNAs were stored at −80 °C.

### 5.10 sgRNA

We used sgRNAs that had previously been demonstrated to be efficient for base editing in mice.^42,43,65^ Guide RNAs targeting the Pcsk9 gene and Dnmt1 gene were chemically synthesized by GenScript. The modified synthetic sgRNAs contained 2′-*O*-methyl analogs on the first and last three bases and 3′phosphorothioate linkages between the first three and last two bases. Protospacer sequence of sgDNMT1 and sgPCSK9 is shown in Table S2.

### 5.11 Next-Generation Sequencing

To assess editing rates, DNA was extracted from organs using a Qiagen DNEasy Blood and Tissue kit. For liver analysis, the liver lobes were cut into small pieces and mixed before sampling. Genomic DNA was amplified by PCR using primers specific to the target genomic sites containing adapters for Illumina sequencing. Dnmt1 was amplified using the primers:

Forward: 5′-ACACTC­TTTCCC­TACACG­ACGCTC­TTCCGA­TCTNNN­NCCTTC­GGGCAT­AGCA­TGGTC −3′

Reverse: 5′-TGGAGT­TCAGAC­GTGTGC­TCTTCC­GATCTT­ATATGC­CTCGGC­ATCG­GTCC-3′

Pcsk9 was amplified using the primers:

Forward: 5′-ACACTC­TTTCCC­TACACG­ACGCTC­TTCCGA­TCTNNN­NGCTGC­TGTTGC­TGCTA­CTGT-3′

Reverse: 5′-TGGAG­TTCAGA­CGTGTG­CTCTTC­CGATCT­CAAGGT­GCAGCT­GAGC­ACTA −3′

The PCR reaction contained 0.4 μM of each primer, 1× Q5 High-Fidelity Master Mix (New England Biolabs) or 1× Phusion U Multiplex PCR Master Mix (Thermo Fisher Scientific), 100 ng genomic DNA, and water to a final volume of 25 μL. The following PCR program was used: step 1, 95 °C for 2 min; step 2, 98 °C for 10 s; step 3, annealing for 30 s; step 4, 72 °C for 2 min; step 5, repeat steps 2–4 for a total of 30 cycles; step 6, 72 °C for 2 min; and step 7, 4 °C. After Illumina barcoding, PCR products were purified by electrophoresis with a 2% agarose gel using a QIAquick Gel Extraction Kit (Qiagen), eluting with 30 μL water. DNA concentration was quantified using a Qubit dsDNA High Sensitivity Assay Kit (Thermo Fisher Scientific) and by qPCR using the KAPA Library Quantification Kit (Roche) before being sequenced on an Illumina MiSeq instrument (single-end read, 250–300 cycles) according to the manufacturer’s protocols. Alignment of fastq files and quantification of editing frequency were performed using CRISPResso2 in batch mode with a window width of 20 nucleotides. The total editing percentage was quantified by using the percentage of reads with a G at the target adenine position after alignment.

### 5.12 Quantification of PCSK9 Protein Levels

At definite time intervals, 50–100 μL of blood was collected from each mouse by submandibular bleed. Plasma was collected and stored at −80 °C until analysis. The plasma was diluted 500x, and the levels of PCSK9 were determined using the ELISA kit according to the manufacturer’s protocol. After the termination of the experiment, livers were extracted and cut into small pieces. A 100 mg tissue was mixed with 400 μL reporter lysis buffer (Promega) and homogenized for 20 at 6 m/s using MP Biomedical FastPrep-24 5G Homogenizer. The tubes were then spun for 5 min at 8000× *g*. The supernatant was diluted 10× for the evaluation of PCSK9 protein levels using the same ELISA kit.

### 5.13 Quantification of Plasma Cholesterol

At definite time intervals, 50–100 μL blood was collected from each mouse by submandibular bleed. Plasma was collected and stored at −80 °C until analysis. Total cholesterol concentration was quantified using the Cholesterol E kit. Briefly, 2 μL plasma was mixed with 200 μL of color reagent and incubated at 37 °C for 10 min. Plasma concentrations were then determined using a standard cholesterol solution.

### 5.14 Statistical Analysis

The statistical significance between the TriGalNAc-conjugated and unconjugated LNPs was determined using multiple two-tailed, unpaired *t* tests. Multiple comparisons among three or more groups were performed using one-way or two-way ANOVA, followed by Tukey’s post hoc test unless indicated otherwise. Two-tailed, Spearman’s rank correlation coefficient was used to assess monotonic relationships. A statistically significant difference was set at *p* < 0.05. ns: nonsignificant. *P < 0.05. **P < 0.01. ***P < 0.001. ****P < 0.0001.

## Supplementary Material



## Notes

Table listing all LNPs compositions and lipid concentrations; IVIS images of all animals tested in the study; The relationship between PEG-lipid structure and the synergistic enhancement in hepatic mRNA delivery; Tissue accumulation of Cy5-labeled mRNA; Flow cytometric analysis of mCherry expression in hepatocytes; Effect of ligand spacer PEG length on hepatic mRNA delivery; In vitro cellular uptake and luciferase expression analysis; Quantification of ApoE binding; Extended panel of CryoTEM images; IgM production following LNP intravenous injections (PDF)

## Abbreviations

ABE: adenine base editor
ApoE: apolipoprotein E
ASGPR: asialoglycoprotein receptor
DiD: 1,1′-dioctadecyl-3,3,3′,3′-tetramethylindodicarbocyanine, 4-chlorobenzenesulfonate
DMG: dimyristoylglycerol
DNMT1: DNA methyltransferase 1
DODAP: 1,2-dioleoyl-3-dimethylammonium-propane
DSPE: 1,2-distearoyl-*sn*-glycero-3-phosphoethanolamine
DSPC: 1,2-distearoyl-*sn*-glycero-3-phosphocholine
LDLR: low-density lipoprotein receptor
LNP: lipid nanoparticle
PCSK9: proprotein convertase subtilisin/kexin type 9
PEG: polyethylene glycol
RES: reticuloendothelial system
sgRNA: single guide RNA
TriGalNAc: triantennary *N*-Acetylgalactosamine
cryoTEM: cryogenic transmission electron microscope
