# Supplementary material for: Boosting Ligand-Mediated Hepatic mRNA Delivery and Gene Editing Through Coordinated Pairing of Polyethylene Glycol-Modified Lipids
Source: ACS Nano. 2026 Sep 8;20(37):25642–59. doi: 10.1021/acsnano.6c10855 (PMC13602997; doi:10.1021/acsnano.6c10855)
Supplement: Supplementary file 1 [file nn6c10855_si_001.pdf]

Supporting Information

Boosting ligand-mediated hepatic mRNA delivery and gene editing through coordinated pairing of polyethylene glycol-modified lipids

Saed Abbasi[1,2,‡], *Kimberly M. Bockley*[1,3,‡], *Johannes Dohr*[4], *Emily Chen*[1,5], *Davell M. Carter*[1,3], *Cynthia A. Berlinicke*[2], *Xizhen Lian*[6,7,8], *Maria Viskadourou*[4], *Emma Burke*[4], *Laura M. Ensign*[*1-3,5,9], *Gregory A. Newby*[4,7,9] *and Justin Hanes*[*1-3,5, 7, 9]

[1]Center for Nanomedicine at the Wilmer Eye Institute, Johns Hopkins University School of Medicine, Baltimore, MD 21231, USA

[2]Department of Ophthalmology, Johns Hopkins University School of Medicine, Baltimore, MD 21231, USA

[3]Department of Physiology, Pharmacology & Therapeutics, Johns Hopkins University School of Medicine, Baltimore, MD 21231, USA

[4]Department of Genetic Medicine, Johns Hopkins University, Baltimore, MD 21205, USA

[5]Department of Chemical and Biomolecular Engineering, Johns Hopkins University, Baltimore, MD 21231, USA

[6]Department of Materials Science and Engineering, Johns Hopkins University, Baltimore, MD 21231, USA

[7]Institute for NanoBioTechnology, Johns Hopkins University, Baltimore MD 21231, USA

[8]Department of Pediatrics, Johns Hopkins University School of Medicine, Baltimore MD 21231, USA

[9]Department of Biomedical Engineering, Johns Hopkins University, Baltimore, MD 21231, USA

[‡]These authors equally contributed to this work

*Correspondence to:

Saed Abbasi: sabbasi5@jhmi.edu

Laura M. Ensign: lensign@jhmi.edu

Justin Hanes: hanes@jhmi.edu

Supplementary Methods

Quantification of ApoE binding

fLuc mRNA-loaded LNPs were incubated in equal volumes of mouse plasma (140 µL each) at 37 °C for 30 min to allow for protein corona formation. Each sample was prepared in triplicate. mRNA-LNPs were collected by ultracentrifugation (100,000 × g, 4 °C, 1 h), as previously described[1]. The supernatant was removed carefully and discarded. The samples were resuspended in 1 mL of 1× PBS and spun at 100,000 × g, 4 °C, and 15 min. The washing step was repeated in a total of 3 times. After the last wash, the supernatant was removed carefully and discarded, and the pellet was resuspended in 50 µL of RIPA buffer containing protease inhibitors. Protein concentrations were quantified using a Qubit assay (Thermo) to add equal amounts of proteins into SDS-PAGE. Proteins were then transferred to PVDF membranes (Millipore). Before blocking, the total protein amount for each lane was stained (No-Stain Protein Labeling Reagent, Invitrogen) and imaged. Membranes were blocked with antibody blocking buffer for 1 h (WesternBreeze, Invitrogen Life Technologies), then incubated with primary antibody against mouse ApoE (1:1000 dilution, Cell Signaling Technology). After washing, membranes were treated with an alkaline phosphatase-conjugated secondary antibody for 1 h at room temperature (WesternBreeze, Invitrogen Life Technologies). The total protein amount as well as the protein bands were visualized using Bio Rad ChemiDocMP.

Quantification of anti-PEG IgM

Seven days after a single injection of fLuc mRNA-LNPs at a dose of 7.5 µg mRNA in BALB/c mice, blood from the inferior vena cava was collected, processed to plasma, and stored in -80 °C until analysis. Anti-PEG IgM levels were quantified by the ELISA kit according to the manufacturer's protocol. Anti-PEG IgM levels in the untreated animals was reported as zero since the absorbance was below the detection limit of the ELISA kit.

Flow Cytometry

BALB/c mice were intravenously injected with mRNA-LNPs at a dose equivalent 7.5 µg mCherry mRNA. After one day, mice were scarified the livers were extracted and treated with 2 mg/mL liberase (Sigma Aldrich, 5401119001) for 30 min with constant rotation at 30 °C. The livers were then passed through a 60 µm cell strainer and resuspended in 3 mL ACK lysis buffer for 3 min to remove red blood cells. The reaction was then neutralized by diluting the suspension in 10 volumes of flow cytometry buffer (0.5% w/v BSA in PBS). Cells were then spun and concentrated to 10^6 cells per 200 µL and incubated with Zombie aqua Live/Dead (1:1000), PerCP Cy5.5 CD45 (1:400), and FITC CD31 (1:50) for 1 h. The cells were then analyzed using a Sony flow cytometer, and the percentage of hepatocytes ($CD45^{-}CD31^{-}$) that are positive for mCherry expression was quantified as previously described[2].

Supplementary Tables and Figures

Supplementary Table S1. Lipid composition and physicochemical characteristics of mRNA-LNPs formulated in this study.

#	LNP ID	mRNA type	Chole-Sterol (mol%)	DSPC (mol%)	Ionizable cationic lipid		PEG-lipid				DSPE-PEG2000-TriGalNAc (mol%)	Z-average size (nm)	PDI
					Type	Mol %	Type	Tail length	Linker type	Mol %			
1	LNP1	fLuc	38.5	10	MC3	50	DMG-PEG2000	C14	Glycerol	1.5	0	102.3	0.22
2	LNP2	fLuc	38.5	10	MC3	50	DMG-PEG2000	C14	Glycerol	1.5	0.1	96.2	0.25
3	LNP3	fLuc	37	10	MC3	50	DMG-PEG5000	C14	Glycerol	3	0	163.8	0.20
4	LNP4	fLuc	37	10	MC3	50	DMG-PEG5000	C14	Glycerol	3	0.1	179.5	0.26
5	LNP5	fLuc	37	10	MC3	50	DMG-PEG2000	C14	Glycerol	3	0	125.3	0.18
6	LNP6	fLuc	37	10	MC3	50	DMG-PEG2000	C14	Glycerol	3	0.1	129.1	0.18
7	LNP7	fLuc	37	10	MC3	50	DMG-PEG500	C14	Glycerol	3	0	155.1	0.16
8	LNP8	fLuc	37	10	MC3	50	DMG-PEG500	C14	Glycerol	3	0.1	152.7	0.12
9	LNP9	fLuc	37	10	MC3	50	DMPE-PEG2000	C14	Phosphate	3	0	181.0	0.06
10	LNP10	fLuc	37	10	MC3	50	DMPE-PEG2000	C14	Phosphate	3	0.1	160.5	0.04
11	LNP11	fLuc	37	10	MC3	50	DMPE-PEG350	C14	Phosphate	3	0	159.9	0.19
12	LNP12	fLuc	37	10	MC3	50	DMPE-PEG350	C14	Phosphate	3	0.1	181.0	0.06
13	LNP13	fLuc	37	10	MC3	50	DSG-PEG2000	C18	Glycerol	3	0	108.9	0.12
14	LNP14	fLuc	37	10	MC3	50	DSG-PEG2000	C18	Glycerol	3	0.1	116.1	0.18
15	LNP15	fLuc	37	10	MC3	50	DSG-PEG500	C18	Glycerol	3	0	153.6	0.05
16	LNP16	fLuc	37	10	MC3	50	DSG-PEG500	C18	Glycerol	3	0.1	146.5	0.06
17	LNP17	fLuc	37	10	MC3	50	DSPE-PEG5000	C18	Phosphate	3	0	117.8	0.15
18	LNP18	fLuc	37	10	MC3	50	DSPE-PEG5000	C18	Phosphate	3	0.1	122.0	0.39
19	LNP19	fLuc	37	10	MC3	50	DSPE-PEG2000	C18	Phosphate	3	0	104.1	0.26
20	LNP20	fLuc	37	10	MC3	50	DSPE-PEG2000	C18	Phosphate	3	0.1	81.6	0.25
21	LNP21	fLuc	37	10	MC3	50	DSPE-PEG350	C18	Phosphate	3	0	150.2	0.07
22	LNP22	fLuc	37	10	MC3	50	DSPE-PEG350	C18	Phosphate	3	0.1	126.2	0.13
23	LNP23	fLuc	38.5	10	MC3	50	DSPE-PEG350	C18	Phosphate	1.5	0	171.0	0.07
24	LNP24	fLuc	38.5	10	MC3	50	DSPE-PEG2000	C18	Phosphate	1.5	0	99.6	0.06
25	LNP25	fLuc	38.5	10	MC3	50	DSPE-PEG5000	C18	Phosphate	1.5	0	71.5	0.15

26	LNP26	fLuc	38.5	10	DODAP	50	DMG-PEG2000	C14	Glycerol	1.5	0	96.5	0.29
27	LNP27	fLuc	38.5	10	DODAP	50	DMG-PEG2000	C14	Glycerol	1.5	0.1	122.4	0.36
28	LNP28	fLuc	37	10	DODAP	50	DSPE-PEG350	C18	Phosphate	3	0	128.6	0.11
29	LNP29	fLuc	37	10	DODAP	50	DSPE-PEG350	C18	Phosphate	3	0.1	126.3	0.13
30	LNP30	fLuc	37	10	4A3-SC8	50	DSPE-PEG350	C18	Phosphate	3	0	676.8	0.86
31	LNP31	fLuc	37	10	4A3-SC8	50	DSPE-PEG350	C18	Phosphate	3	0.1	450.7	0.59
32	LNP1	ABE8e: sgDNMT1 (2:1)	38.5	10	MC3	50	DMG-PEG2000	C14	Glycerol	1.5	0	108.1	0.19
33	LNP22	ABE8e: sgDNMT1 (2:1)	37	10	MC3	50	DSPE-PEG350	C18	Phosphate	3	0.1	111.6	0.11
34	LNP1	ABE8e: sgPCSK9 (2:1)	38.5	10	MC3	50	DMG-PEG2000	C14	Glycerol	1.5	0	142.4	0.12
35	LNP22	ABE8e: sgPCSK9 (2:1)	37	10	MC3	50	DSPE-PEG350	C18	Phosphate	3	0.1	151.1	0.09

Supplementary Table S2. Protospacer sequence for sgRNAs used in this study

**sgRNA**	**Protospacer (5′ to 3′)**
sgDNMT1	AACAGCTCTGAACGAGACCC
sgPCSK9	CCCATACCTTGGAGCAACGG

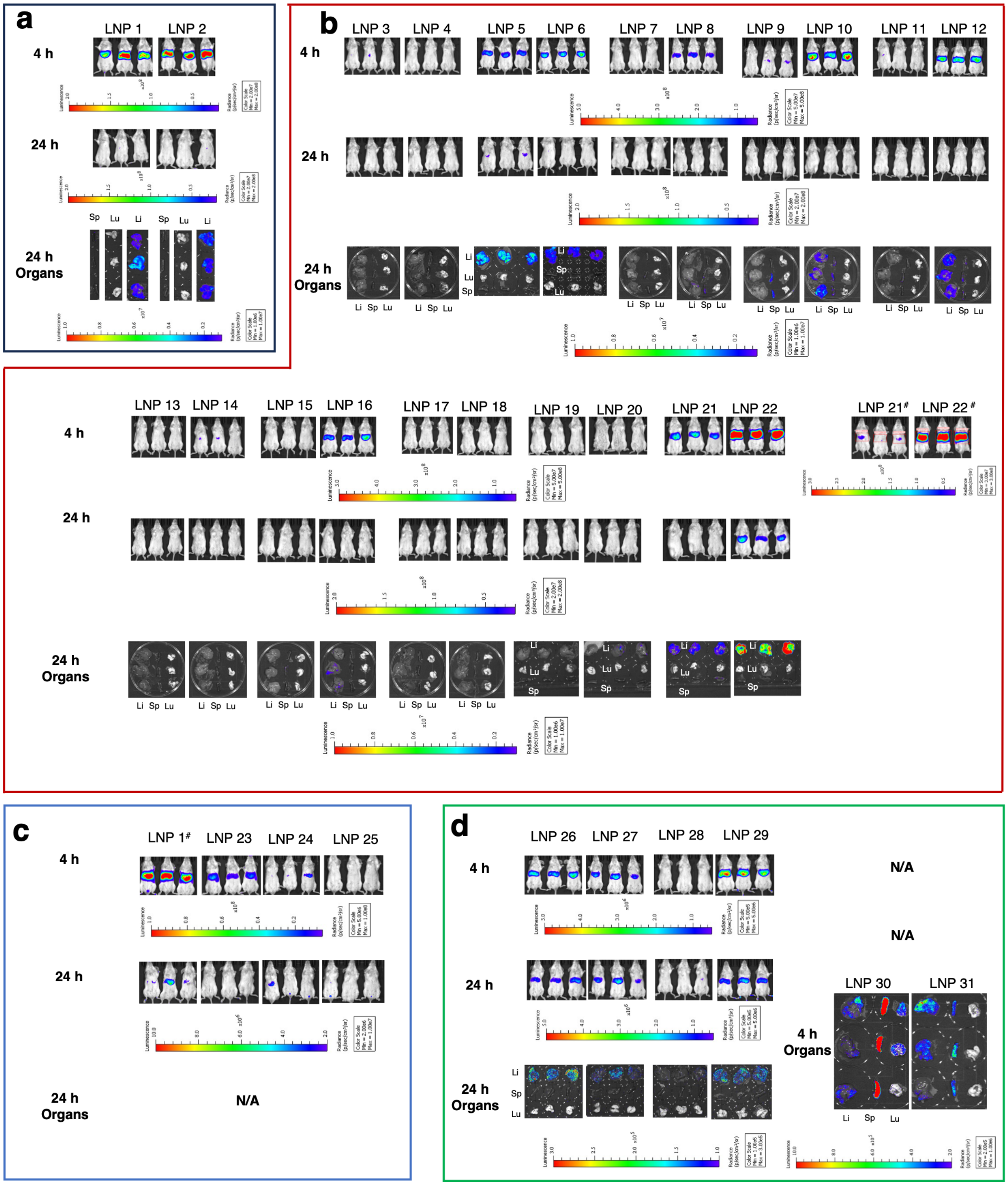

Supplementary Figure S1. IVIS images of all animals tested in the study. Evaluations of luciferase expression in animals treated with LNPs 1-31 as presented in main Figure 1 (a), Figure 2 (b), Figure 4 (c), and Figure 5 (d). Li: liver. Sp: spleen. Lu: lung. n=3 mice. #Indicates an independently replicated experiment performed in an additional group of n = 3 mice.

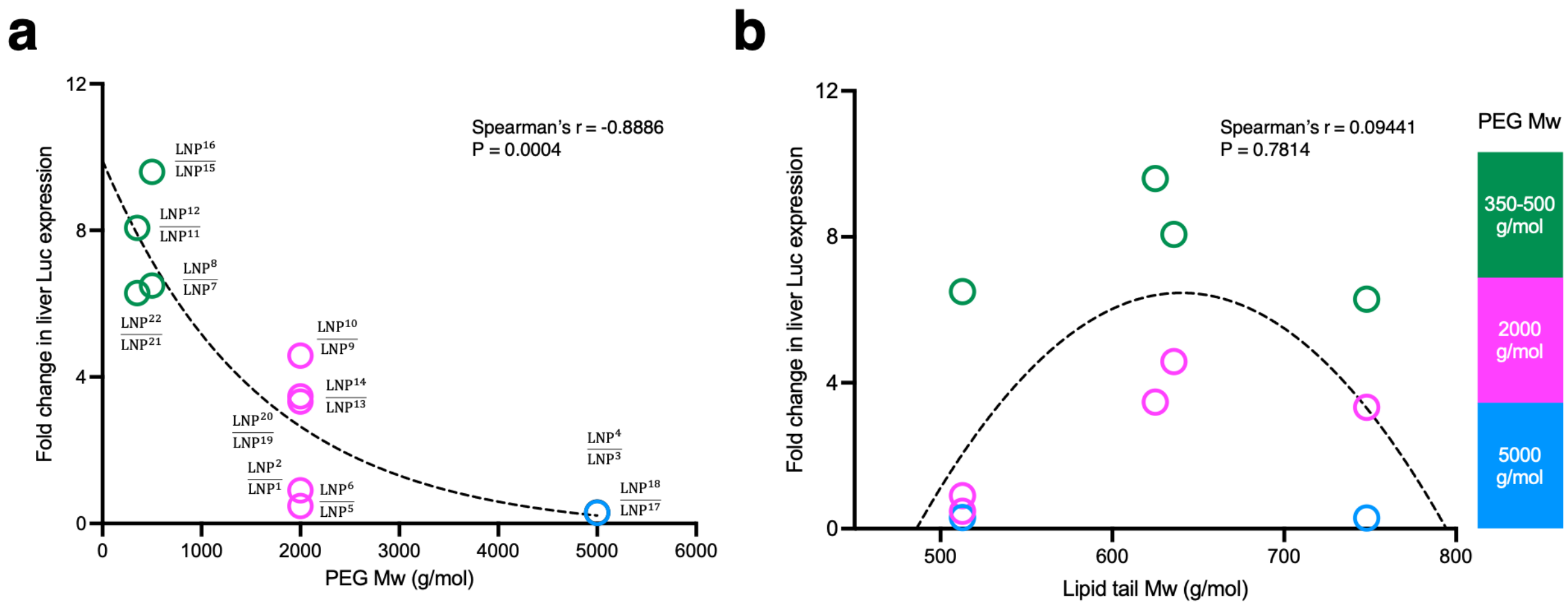

Supplementary Figure S2. The relationship between PEG-lipid structure and the synergistic enhancement in hepatic mRNA delivery induced by pairing the TriGalNAc-lipid with a PEG-lipid. (a) Impact of the PEG chain molecular weight of the PEG-lipid on the enhancement of TriGalNAc-mediated liver delivery. **(b)** Impact of the lipid tail molecular weight of the PEG-lipid on the enhancement of TriGalNAc-mediated liver delivery. Fold-changes were calculated by dividing the luciferase expression in extracted livers of mice treated with TriGalNAc-conjugated LNPs by that of mice treated with the unconjugated counterparts 24 hours post-injection. Correlation was assessed using a two-tailed, Spearman's rank correlation coefficient.

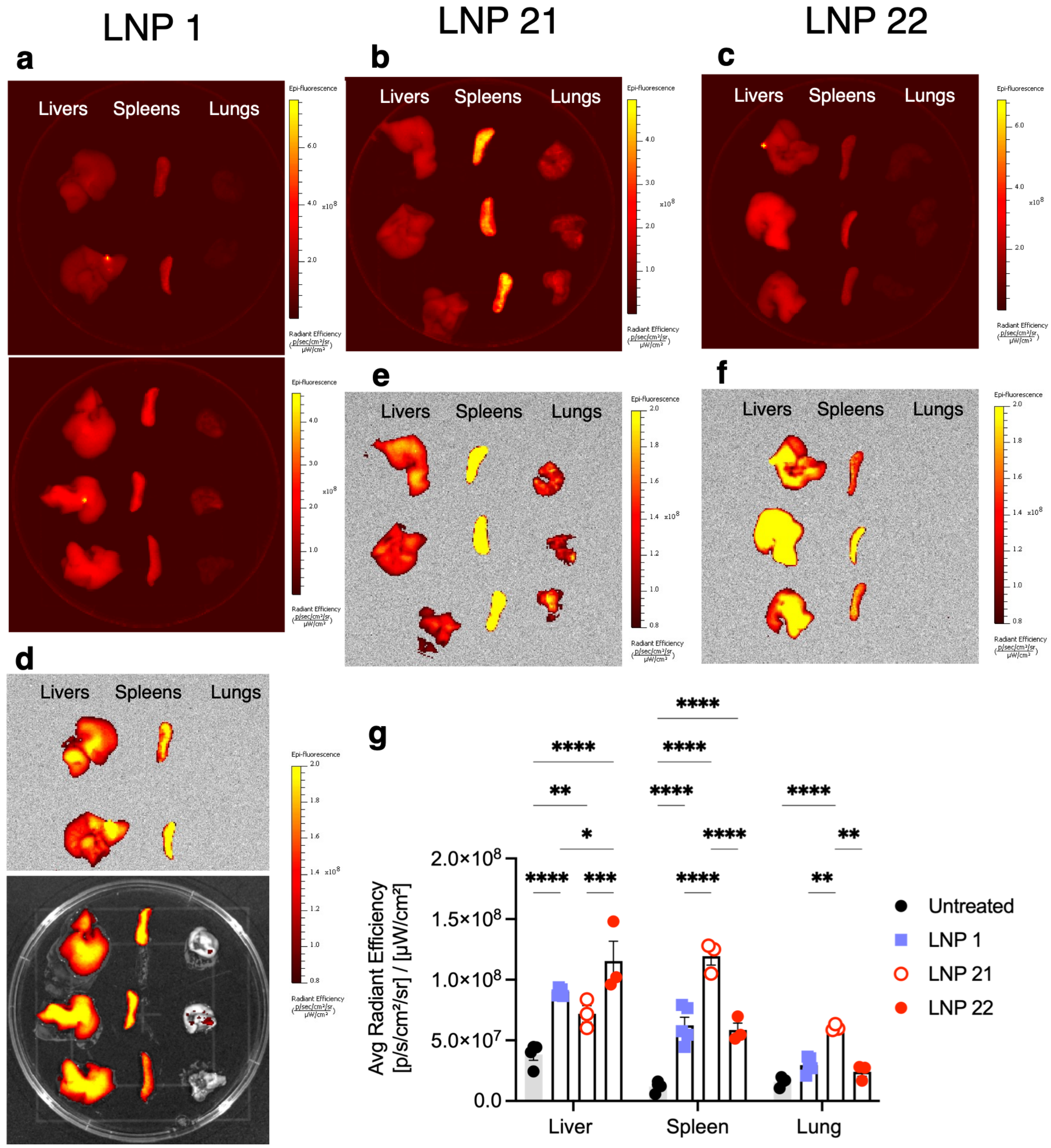

Supplementary Figure S3. Quantification of LNPs containing Cy5-labelled mRNA in mouse organs. (a, d) LNP 1**. (b, e)** LNP 21. **(c, f)** LNP 22. **(g)** Quantification of average radiant efficiency in each organ including untreated organs receiving no treatment. **(a-c)** IVIS fluorescence images displaying the entire dynamic range of detected signal intensities present in the selected image. **(d-f)** IVIS images showing organs in a-c but after normalization to a single intensity scale across the three treatment groups. BALB/c mice were injected with 7.5 µg Cy5-labelled fLuc mRNA loaded into LNPs by tail vein injection. One hour after injection, mice were sacrificed and organs were imaged at excitation λ = 620 nm and emission λ = 670 at 10 sec exposure time. The data represent the mean ± SEM (n=3, except LNP 1, n=5). Statistical significance was calculated using two-way ANOVA followed by Tukey's post-hoc analysis. ns: nonsignificant. *P<0.05. **P<0.01. ***P<0.001. ****P<0.0001.

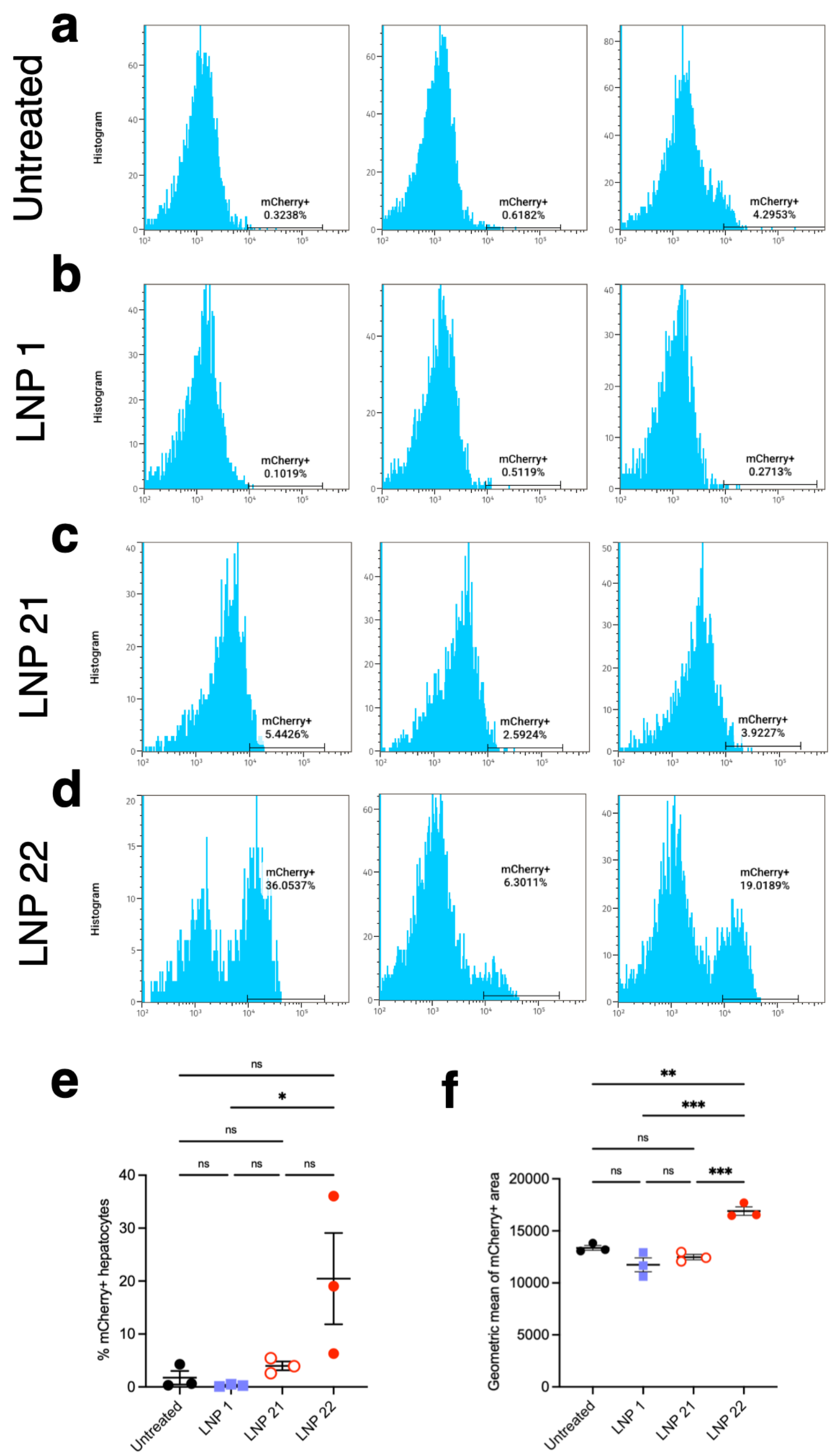

Supplementary Figure S4. Evaluation of mCherry expression in liver hepatocytes. (a) Untreated control. **(b)** LNP 1. **(c)** LNP 21. **(d)** LNP 22. Each histogram represents mCherry expression in the liver hepatocytes in each of the three mice per group. Hepatocytes were identified by negative staining for CD31 and CD45. BALB/c mice were injected 7.5 µg mCherry mRNA loaded into LNPs by tail vein injection. After one day, mice were sacrificed and livers were process by flow cytometry. **(e)** The percentage of hepatocytes positive for mCherry expression in untreated and treated livers. **(f)** The geometric mean of the mCherry positive area as calculated using the FlowJo software. The data represent the mean ± SEM (n=3 mice). Statistical significance was calculated using one-way ANOVA followed by Tukey's post-hoc analysis. ns: nonsignificant. *P<0.05. **P<0.01. ***P<0.001. ****P<0.0001.

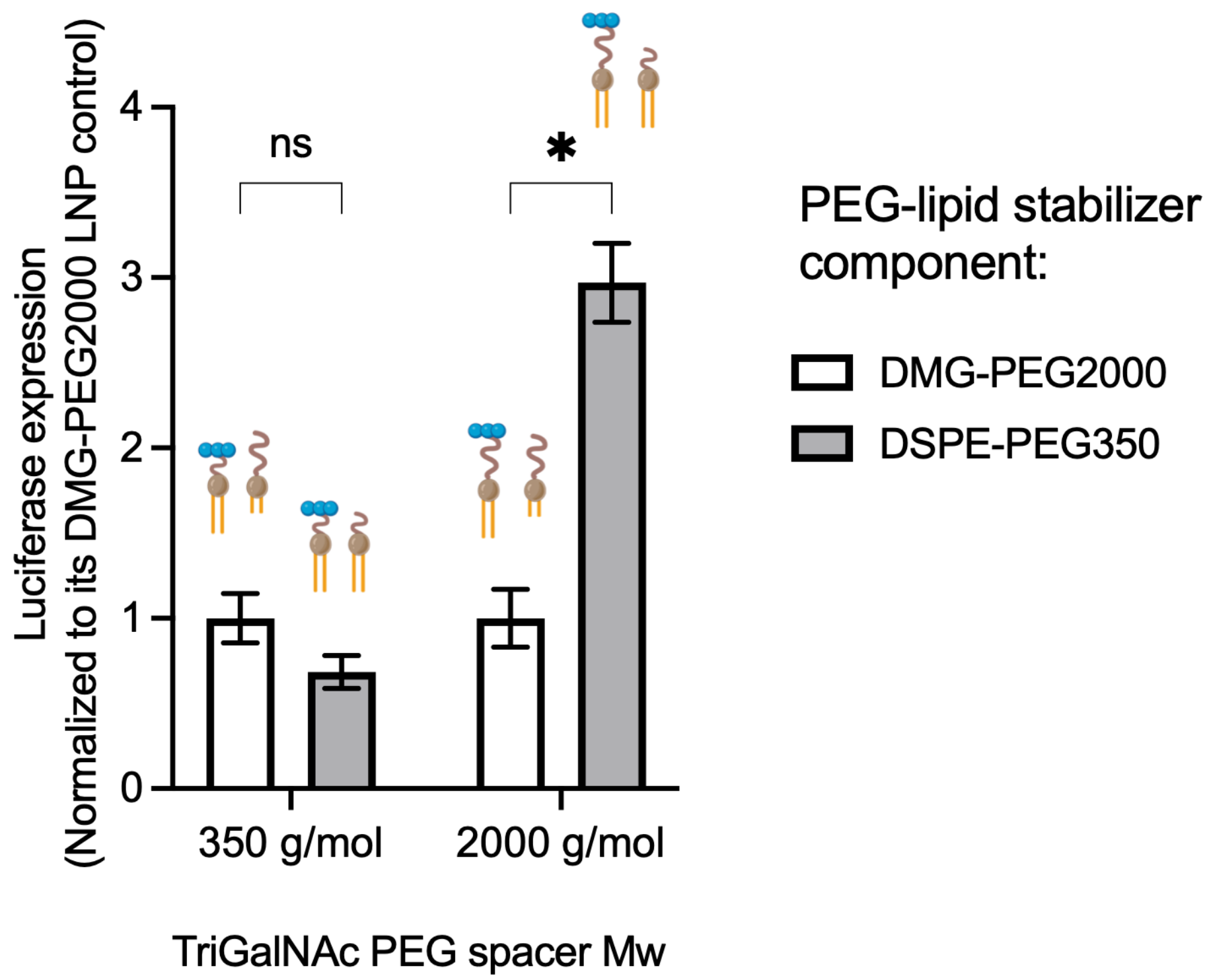

Supplementary Figure S5. Impact of the TriGalNAc ligand spacer PEG length on the synergistic enhancement in hepatic mRNA delivery. TriGalNAc-lipid concentration was fixed at 0.1 mol%. In one group, the TriGalNAc ligand spacer PEG was fixed at 350 g/mol, while in the other group, the PEG spacer was fixed at 2000 g/mol. In each group, the effect of replacing 1.5 mol% DMG-PEG2000 with 3 mol% DSPE-PEG350 on boosting luciferase expression in the liver was evaluated. Luciferase expression in the liver was measured 4 h post intravenous injection of 7.5 µg fLuc mRNA in BALB/C mice, as quantified by whole-body imaging using IVIS. The data represent the mean ± SEM (n=3). Statistical significance was calculated using unpaired t-tests. ns: nonsignificant. *P<0.05. **P<0.01. ***P<0.001. ****P<0.0001.

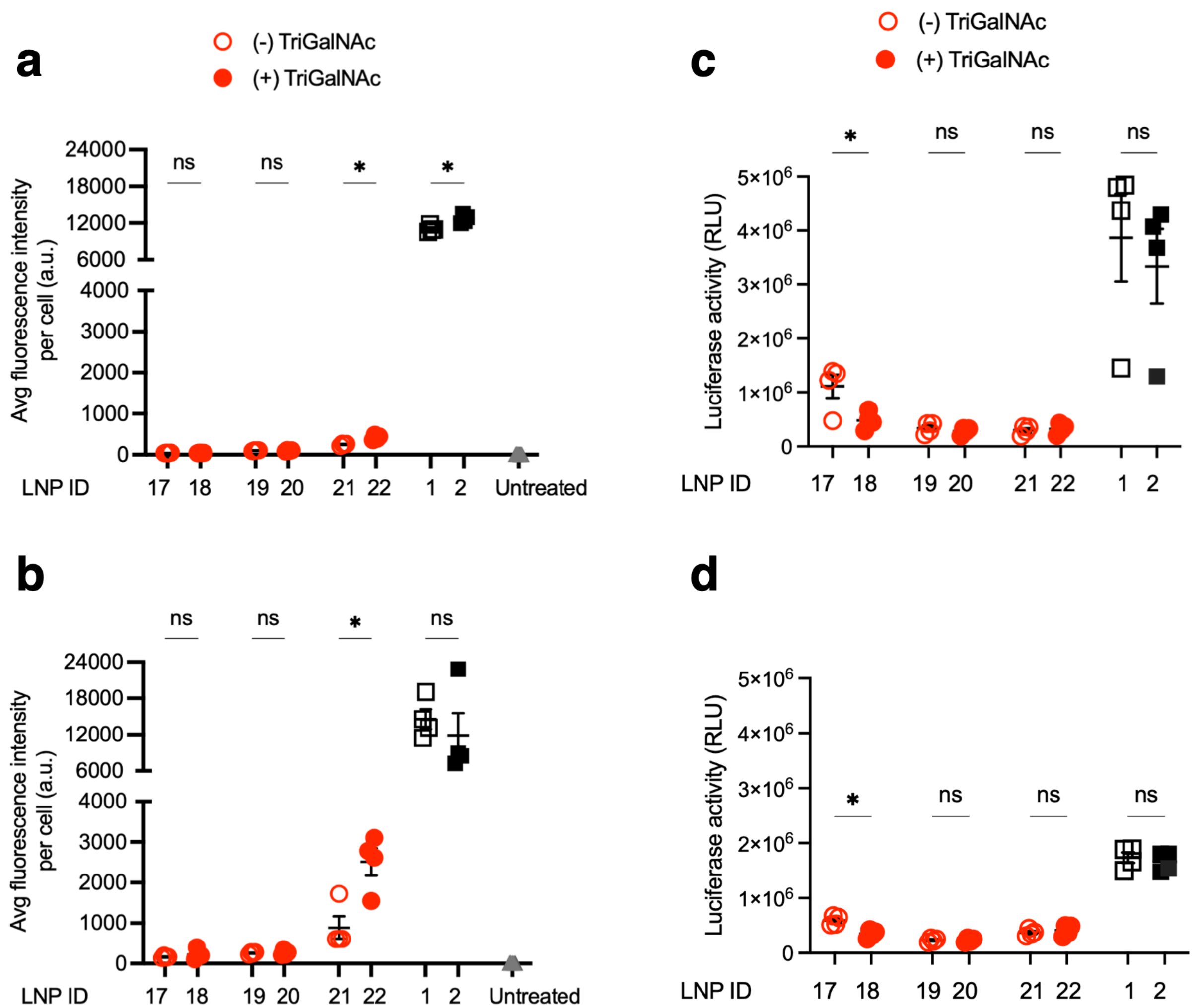

Supplementary Figure S6. In vitro cellular uptake and luciferase expression analysis. Hep G2 cells were incubated with DiD-labelled LNPs at 25 ng Fluc mRNA per well and further incubated for 24 h in in the presence of 10% fetal bovine serum (FBS) (**a, c**) or absence of serum (**b, d**). (**a, b**) Quantification of the average DiD signal intensity per cell in each well. The data represent the mean ± SEM (n=4 wells). (**c, d**) Quantification of luciferase expression in cell lysates after capturing the fluorescence images. The data represent the mean ± SEM (n=4 wells). Statistical significance was calculated using two-tailed, unpaired t-tests between the TriGalNAc-conjugated and unconjugated LNPs. ns: nonsignificant. *P<0.05.

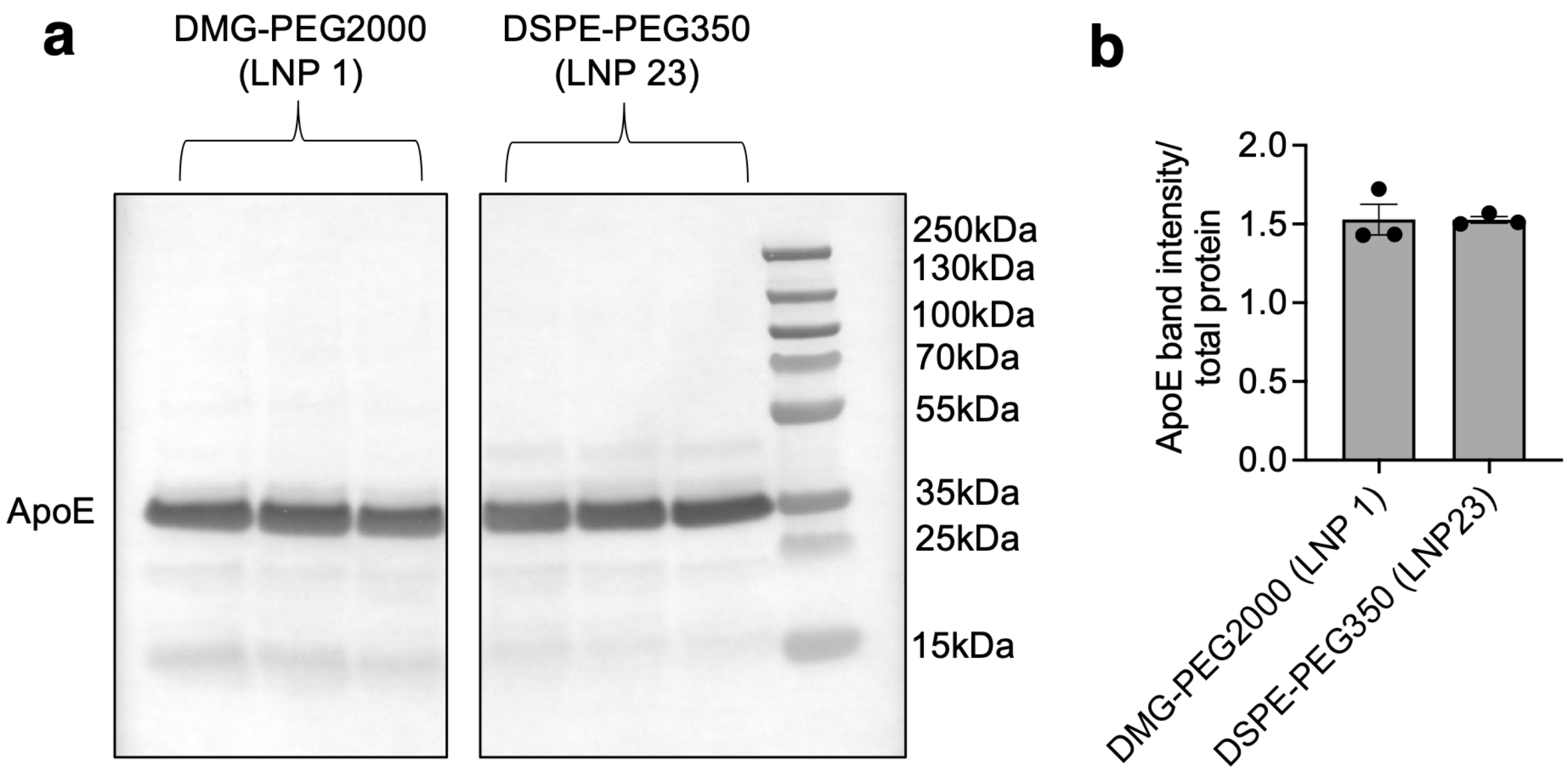

Supplementary Figure S7. Quantification of ApoE binding on mRNA-LNP surfaces. TriGalNAc-unconjugated LNPs were prepared at 1.5 mol% PEG -lipid. **(a)** Western blot images. **(b)** Quantification of band intensity normalized to total protein stain. LNPs 1 and 23 were incubated with mouse plasma at 37 °C for 30 min to allow protein corona formation (n=3 independent replicates). Protein-bound particles were collected by ultracentrifugation, washed three times, and resuspended in RIPA buffer containing protease inhibitors. Samples were subjected to SDS-PAGE followed by Western blotting using an antibody against mouse ApoE. The data shown represent three independent replicates per group, each replicate in a separate lane. All samples were run on the same gel, but LNP 1 and LNP 23 groups were applied in nonadjacent lanes.

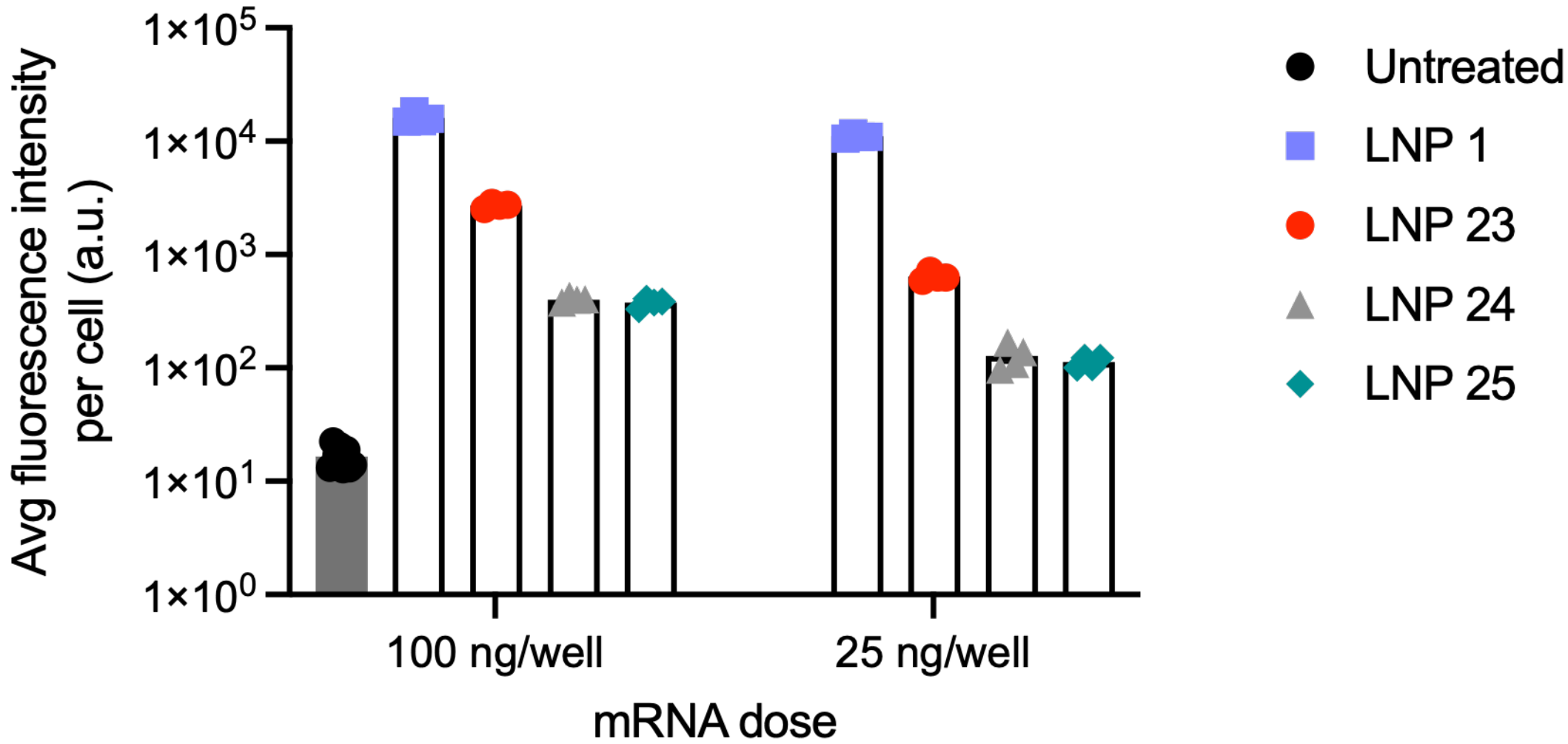

Supplementary Figure S8. Effect of PEG-lipid stabilizer structure on cellular uptake in vitro. Hep G2 cells were incubated with DiD-labelled LNPs containing Fluc mRNA at 25 ng or 100 ng mRNA per well and further incubated for 24 h in in the presence of 10% fetal bovine serum (FBS). Quantification of the average DiD signal intensity per cell in each well is presented. The data represent the mean ± SEM (n=4 wells).

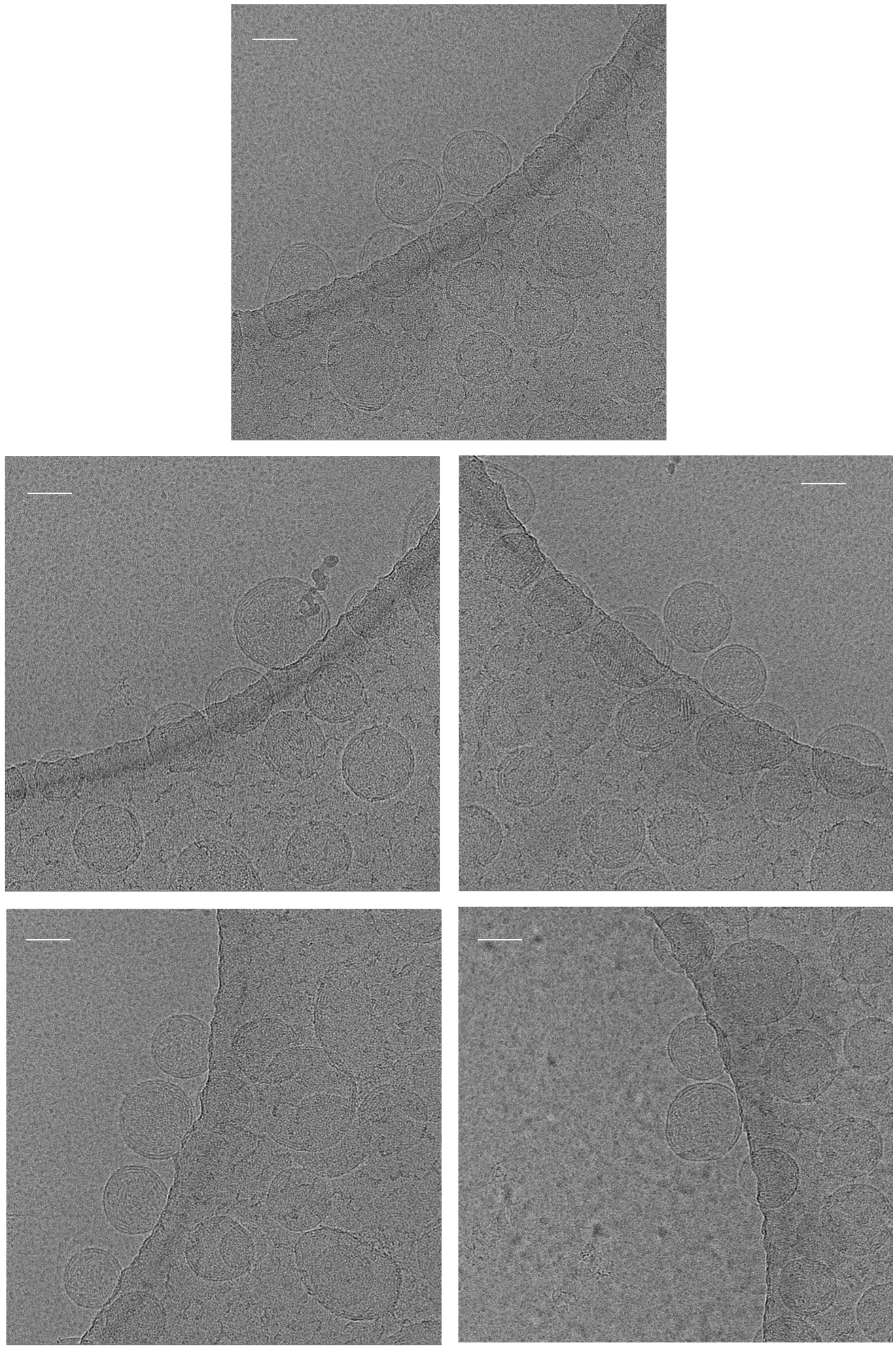

Supplementary Figure S9. Extended panel of CryoTEM images representative of LNP 22. Scale bar is 50 nm.

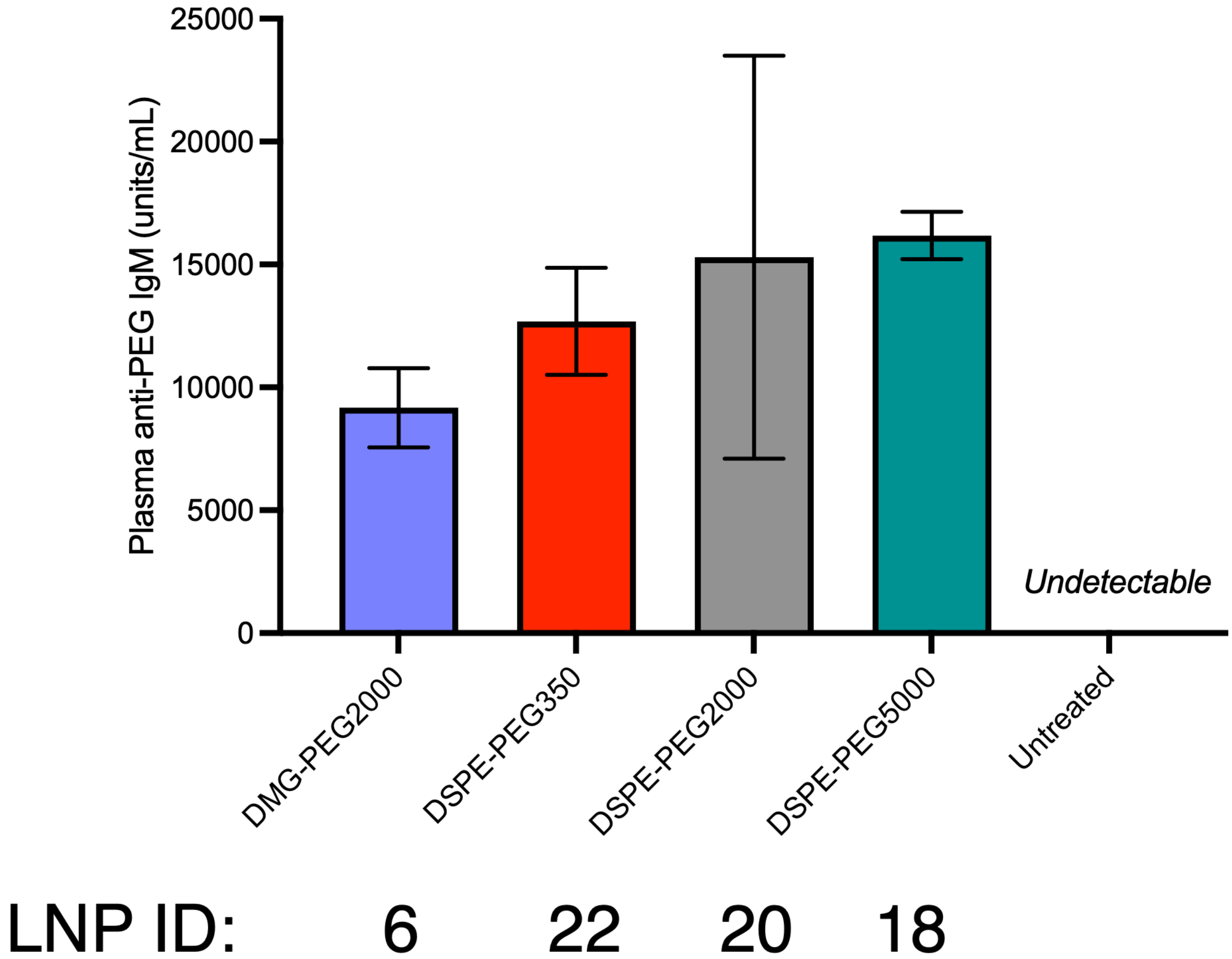

Supplementary Figure S10. Production of anti-PEG IgM in mouse plasma. BALB/c mice were injected with fLuc mRNA-loaded LNPs at 7.5 µg mRNA per mouse, followed by IgM quantification in blood plasma obtained on day 7 post-injection using ELISA. All LNPs contained 3 mol% PEG-lipid and 0.1 mol% DSPE-PEG2000-TriGalNAc. The data represent the mean ± SEM (n=4).

References

1. Qi, Y.; Fu, Y.; Wu, X.; Chen, Z.; Tang, Y.; Ao, H.; Zhu, H.; Zou, C.; Sun, B.; Zheng, C.; Wang, Z.; Hou, J.; Shi, Y.; Jin, Z.; Wang, F.; Guo, S., Flower-Shaped Lipid Nanoparticles Evade Apolipoprotein E-Mediated Liver Tropism for Safe and Enhanced Cytokine-Based Cancer Immunotherapy. *ACS Nano* **2025,** *19* (40), 35675-35691.

2. Kim, M.; Jeong, M.; Hur, S.; Cho, Y.; Park, J.; Jung, H.; Seo, Y.; Woo, H. A.; Nam, K. T.; Lee, K.; Lee, H., Engineered ionizable lipid nanoparticles for targeted delivery of RNA therapeutics into different types of cells in the liver. *Sci Adv* **2021,** *7* (9).
